# Geostatistical analysis of disease data: accounting for spatial support and population density in the isopleth mapping of cancer mortality risk using area-to-point Poisson kriging

**DOI:** 10.1186/1476-072X-5-52

**Published:** 2006-11-30

**Authors:** Pierre Goovaerts

**Affiliations:** 1 BioMedware, Inc., Ann Arbor, MI, USA

## Abstract

**Background:**

Geostatistical techniques that account for spatially varying population sizes and spatial patterns in the filtering of choropleth maps of cancer mortality were recently developed. Their implementation was facilitated by the initial assumption that all geographical units are the same size and shape, which allowed the use of geographic centroids in semivariogram estimation and kriging. Another implicit assumption was that the population at risk is uniformly distributed within each unit. This paper presents a generalization of Poisson kriging whereby the size and shape of administrative units, as well as the population density, is incorporated into the filtering of noisy mortality rates and the creation of isopleth risk maps. An innovative procedure to infer the point-support semivariogram of the risk from aggregated rates (i.e. areal data) is also proposed.

**Results:**

The novel methodology is applied to age-adjusted lung and cervix cancer mortality rates recorded for white females in two contrasted county geographies: 1) state of Indiana that consists of 92 counties of fairly similar size and shape, and 2) four states in the Western US (Arizona, California, Nevada and Utah) forming a set of 118 counties that are vastly different geographical units. Area-to-point (ATP) Poisson kriging produces risk surfaces that are less smooth than the maps created by a naïve point kriging of empirical Bayesian smoothed rates. The coherence constraint of ATP kriging also ensures that the population-weighted average of risk estimates within each geographical unit equals the areal data for this unit. Simulation studies showed that the new approach yields more accurate predictions and confidence intervals than point kriging of areal data where all counties are simply collapsed into their respective polygon centroids. Its benefit over point kriging increases as the county geography becomes more heterogeneous.

**Conclusion:**

A major limitation of choropleth maps is the common biased visual perception that larger rural and sparsely populated areas are of greater importance. The approach presented in this paper allows the continuous mapping of mortality risk, while accounting locally for population density and areal data through the coherence constraint. This form of Poisson kriging will facilitate the analysis of relationships between health data and putative covariates that are typically measured over different spatial supports.

## Background

Public health officials frequently use cancer mortality maps to identify areas of excess and their potential causes (e.g. environmental exposure or socio-demographic factors), as well as to guide surveillance and control activities. The interpretation and analysis of those choropleth maps faces three major hurdles: 1) the presence of extreme unreliable rates that typically occur for sparsely populated areas and/or less frequent cancers, 2) the visual bias resulting from the aggregation of health data within administrative units of widely different sizes and shapes, and 3) the mismatch of spatial supports for cancer rates and explanatory variables that prevents their direct use in correlation analysis and often implies an aggregation to the coarser geography. Common pitfalls include devoting unwarranted attention to a few oversized geographical units located in low population density areas or conducting regression analysis at scales that distort the true exposure/response relationship (i.e. ecological fallacy) [1]. What is needed is a coherent spatial methodology that allows both the filtering of the noise caused by the small number problem and the mapping of results as continuous surfaces without subjective administrative boundaries. Creation of isopleth risk maps from aggregated disease rates (i.e. areal data) has been the topic of several papers in the statistical and health science literatures. Geostatistical methods range from straightforward point kriging [2-5] to complex random field models that require distributional assumptions and computationally intensive parameter estimation using Markov Chain Monte Carlo (MCMC) techniques [6,7].

The simplest approach to create isopleth risk maps consists of assigning the aggregated rates to the geographic centroids of the administrative units, which are then interpolated to the nodes of a regular grid using point kriging. In these studies, the noise attached to rates estimated from small populations was either ignored [2], filtered using empirical Bayes smoothers prior to the interpolation [3], or incorporated in the interpolation procedure using binomial [4] or Poisson kriging [5]. When performing point kriging of areal data, the user makes the practical assumption that all the habitants of the administrative unit live at the same location and the measured rate thus refers to this specific location. This assumption is reasonable whenever the units of aggregation are small with respect to the spacing of the interpolation grid. For example, Oliver et al. [4] mapped the risk of childhood cancer over a 2 km grid in the West Midlands of England using binomial kriging and rates measured within small electoral wards. More recently, Ali et al. [5] created dysentery and cholera incidence maps using Poisson kriging and rates measured for patrilineally-related clusters of households. The assumption of point measurement support becomes clearly inappropriate when the administrative units are counties or states [2,3], which calls for specific methods to incorporate the shape and size of those units in the analysis.

In theory, the system of kriging equations is flexible enough to incorporate data measured over a wide variety of supports and to accommodate either punctual or areal (classically termed "blocks" in the geostatistical literature) prediction supports [8,9]. Practical implementation of kriging in presence of irregular geographical units is, however, challenging mainly for the derivation of the areal covariance terms used in the kriging system [10]. Indeed, the covariance between two areas is approximated as the average of the point-support covariance computed between any two points discretizing these two areas. The first challenge is that the point-support semivariogram model cannot be inferred directly from the observations since only areal data are available. Instead, the semivariogram of areal data needs to be modelled, then deconvoluted to yield the point-support model. Second, the discretization of all the measurement and prediction supports, followed by the averaging of covariance values, can be CPU intensive. This partly explains why most geostatistical studies were restricted to the use of punctual data to conduct interpolation at punctual locations or over areas of regular size, such as blocks to be mined or environmental exposure units to remediate.

The geostatistical analysis of areal data has received increasing attention as kriging becomes more popular in the fields of remote sensing, social science, and medical geography. In particular, several authors [11-14] have implemented kriging of areal data to predict punctual or areal values, an approach referred to as "area-to-point" (ATP) or "area-to-area" (ATA) kriging following the terminology in Kyriakidis [14]. ATP kriging allows mapping the variability within geographical units while ensuring the coherence of the prediction so that, for example, disaggregated estimates of count data are non-negative and their sum is equal to the original aggregated count. Gotway and Young [12] applied ATA kriging to the mapping of the number of low birth weight (LBW) babies at the Census tract level, accounting for county-level LBW data and covariates measured over different spatial supports, such as a fine grid of ground-level PM_10 _concentrations or tract population.

Both ATA and ATP kriging have great potential for the analysis of cancer mortality maps, enabling the shape and size of administrative units to be incorporated into the smoothing of choropleth maps and the creation of isopleth risk maps, respectively. These methods must however account for the fact that disease rates are comprised of a numerator and a denominator, leading to data with varying degrees of reliability. Poisson kriging was recently introduced to incorporate varying population sizes and spatial patterns in the processing of cancer mortality data [15,16]. Its implementation was facilitated by the initial assumption that all geographical units are the same size and shape, which allowed the use of geographic centroids in semivariogram estimation and kriging. Another implicit assumption was that the population at risk is uniformly distributed within each unit. Although these assumptions were reasonable for filtering cancer mortality maps that consist of geographical units of similar sizes, point Poisson kriging does not allow a rigorous treatment of the change of support, prohibiting for example the creation of continuous risk surfaces.

It is noteworthy that the increasingly popular full Bayesian modeling approach is overwhelmingly used with the conditional auto-regressive (CAR) model for defining the random effect associated with spatial autocorrelation [17-19]. This computationally convenient choice is reasonable if all geographical entities are of similar size and arranged in a regular pattern but it is not particularly attractive otherwise [6]. There have been several attempts to model the spatial correlation as a function of the distance between centroids of geographical units instead of using the arbitrary neighborhood relationship underlying the CAR model [20]. To quote Kelsall and Wakefield [6], "*This approach is quite simplistic, however, and again does not acknowledge the differing shapes and sizes and relative orientation of the areas*". The same authors describe one of the rare Bayesian models that accounts properly for the spatial support of the data, although it was implemented under the assumption of uniform population distribution within each area. Using semivariograms to model the spatial autocorrelation, they estimated the continuous underlying relative risk function for colorectal cancer mortality in 39 wards of the UK district of Birmingham.

This paper presents the first geostatistical study where the size and shape of administrative units, as well as the population density, is incorporated into the filtering of noisy mortality rates and the mapping of the corresponding risk at a fine scale (i.e. disaggregation). This generalization of the Poisson kriging algorithm introduced by Monestiez et al. [21,22] capitalizes on recent developments in the field of change of support and deconvolution of semivariogram estimated from irregular areal data [10]. The approach is illustrated using age-adjusted lung and cervix cancer mortality rates recorded for white females in two contrasted county geographies: 1) state of Indiana that consists of 92 counties of fairly similar size and shape, and 2) four states in the Western US (Arizona, California, Nevada and Utah) forming a set of 118 counties that are vastly different geographical units. Simulation studies are conducted to compare the performances of the new methodology to simple geostatistical approaches (i.e. point kriging of raw or empirical Bayesian smoothed rates) for the same two county geographies. Performance criteria include the accuracy of the prediction of the underlying risk and the quantification of the attached uncertainty. The lack of realistic spatial models in a full Bayesian approach, coupled with the absence of user-friendly software to implement this type of model [23], prohibited the inclusion of this approach in the comparison study of isopleth mapping algorithms.

## Methods

### Data

The methodology to account for spatial support and population density in Poisson kriging will be illustrated using directly age-adjusted mortality rates for a frequent (lung) and less frequent (cervix) cancer. These data are part of the Atlas of Cancer Mortality in the United States [24] and were downloaded from . The analysis focuses on white female rates recorded over the 1970–1994 period and adjusted using the 1970 population pyramid. Two areas with contrasted county geographies were considered: 1) state of Indiana (Region 1), and 2) four states in the Western US (Arizona, California, Nevada and Utah) that will be referred to as Region 2. The choice of these two specific geography areas was guided by the need to assess performances in two contrasted settings: 1) all geographical units have a fairly similar size and shape, which is the "ideal" situation for methods that ignore the spatial support of the data, and 2) geographical units display a wide range of sizes and shapes, which should favour any approach that implicitly accounts for the spatial support of the data in the analysis. The West coast provided a perfect example for the second type of geography (i.e. set of 118 vastly different counties), while Indiana includes a reasonable number (i.e. 92) of counties that are geometrically fairly similar.

**Figure 1:**
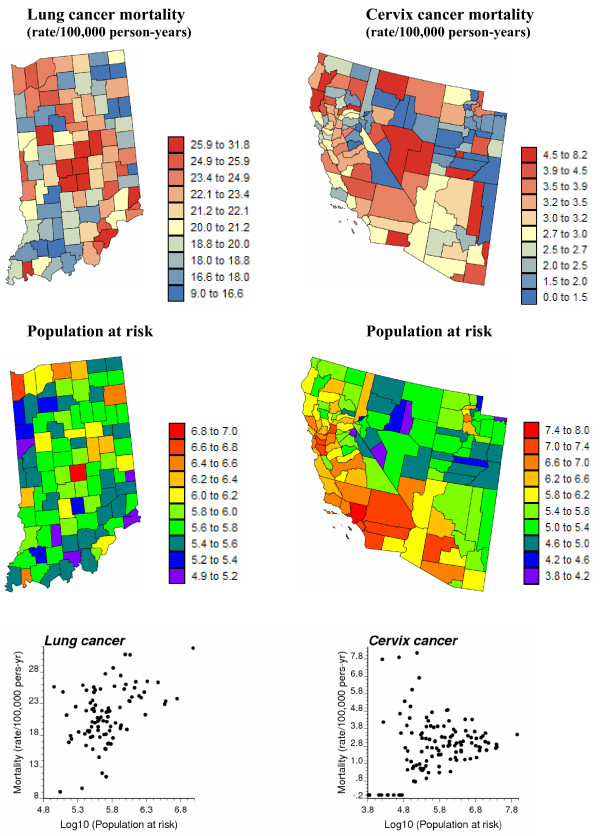
**Geographic distribution of age-adjusted lung and cervix cancer mortality rates and the populations at risk**. For the two top maps, the fill color in each county represents the age-adjusted mortality rates per 100,000 person-years recorded over the period 1970–1994 for white females (class boundaries correspond to deciles of the histogram of rates). The middle maps represent the population at risk which was back-calculated from the rate and count data (lognormal scale). The bottom scatterplots illustrate the larger variance of rates computed from sparsely populated counties, in particular for the least frequent cervix cancer.

Figure 1 (top graphs) shows the spatial distribution of age-adjusted mortality rates per 100,000 person-years for lung cancer in Region 1 and cervix cancer in Region 2. Following the recommendations of several studies on map color schemes [25,26], a double-ended color scheme with 10 equally-weighted classes (i.e. boundaries correspond to deciles of the histogram) was used: a gradient of red is used for rates higher than the median, while a gradient of blue is used for lower rates. The population-weighted average of mortality rates is 23.7 per 100,000 person-years for lung cancer and 2.85 per 100,000 person-years for cervix cancer. For Region 1, the population at risk in each county was computed as: 100,000 × the total number of "lung cancer" deaths over the 1970–1994 period divided by the corresponding age-adjusted mortality rate; both datasets are available on the website of the National Cancer Institute (NCI) of the US (URL as given above). A similar procedure was conducted in Region 2, except that the most frequent breast cancer was used to avoid zero population estimates for a few sparsely populated counties where no cervix cancer mortality was reported during that period. The population sizes are mapped in Figure 1 (middle row) using a rainbow color scheme to avoid any confusion with the rate maps.

A visual inspection of the cervix cancer mortality map conveys the impression that rates are particularly high in the centre of the study area (Nye and Lincoln Counties), as well as in a few Northern California counties. This result must, however, be interpreted with caution since the population is not uniformly distributed across the study area and rates computed from sparsely populated counties tend to be less reliable. This effect, known as "small number problem" [27], is illustrated by the bottom scattergrams in Figure 1. In particular, cervix cancer mortality in excess of 5 deaths per 100,000 person-years is observed only for counties with a 25 year cumulated population smaller than 10^5^. The use of administrative units to report the results (i.e. counties in this case) can also bias the interpretation: had the two counties with the highest rates been much smaller in size, these high values likely would have been perceived as less problematic. The higher frequency of lung cancer mortality, coupled with the narrower range of county sizes in Indiana, makes the interpretation of Region 1 cancer mortality map less hazardous. The highest rate is in fact reported for the most populated county (Marion) whose seat is Indianapolis.

**Figure 2:**
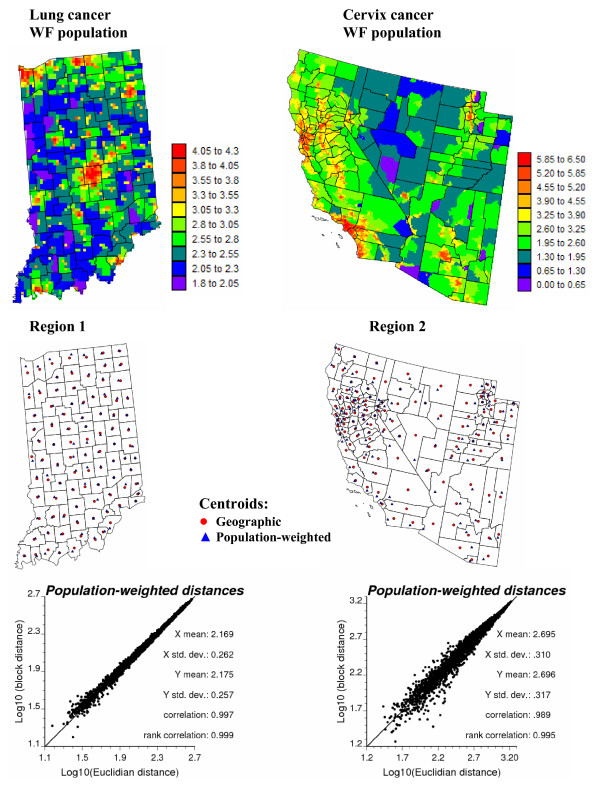
**High-resolution population maps and their use to compute population-weighted distances between counties**. The county-level population estimates of Figure 1 were allocated to a grid of 25 km^2 ^cells according to the 2000 census block level data. The scattergrams plot Euclidian distances between county geographic centroids, depicted by red dots in the middle maps, versus a "block distance" that accounts for the shape of counties and the distribution of the population (Equation 1). Population-weighted centroids are depicted by blue triangles in the middle maps.

To highlight the limitations of collapsing administrative units into their geographic centroids, the spatial distribution of population within each county is mapped at the top of Figure 2. These maps were produced by allocating the county-level population estimates of Figure 1 to a grid of 25 km^2 ^cells discretizing each study area, leading to 3,751 and 48,474 cells in Regions 1 and 2, respectively. The relative proportion of the county-level population within each 25 km^2 ^cell was retrieved from the readily available 2000 census block level data. These population maps illustrate the non-uniform repartition of population, in particular for the vast counties in Region 2. To account for the shape of geographical units and their heterogeneous population density, the distance between any two counties is here estimated as a population-weighted average of Euclidian distances between points discretizing the pair of counties:

Dist(vα,vβ)=1∑s=1Pα∑s′=1Pβn(us)n(us′)∑s=1Pα∑s′=1Pβn(us)n(us′)‖us−us′‖     (Equation 1)
 MathType@MTEF@5@5@+=feaafiart1ev1aaatCvAUfKttLearuWrP9MDH5MBPbIqV92AaeXatLxBI9gBaebbnrfifHhDYfgasaacH8akY=wiFfYdH8Gipec8Eeeu0xXdbba9frFj0=OqFfea0dXdd9vqai=hGuQ8kuc9pgc9s8qqaq=dirpe0xb9q8qiLsFr0=vr0=vr0dc8meaabaqaciaacaGaaeqabaqabeGadaaakeaacqWGebarcqWGPbqAcqWGZbWCcqWG0baDcqGGOaakcqWG2bGDdaWgaaWcbaacciGae8xSdegabeaakiabcYcaSiabdAha2naaBaaaleaacqWFYoGyaeqaaOGaeiykaKIaeyypa0ZaaSaaaeaacqaIXaqmaeaadaaeWbqaamaaqahabaGaemOBa4MaeiikaGccbeGae4xDau3aaSbaaSqaaiabdohaZbqabaGccqGGPaqkcqWGUbGBcqGGOaakcqGF1bqDdaWgaaWcbaGafm4CamNbauaaaeqaaOGaeiykaKcaleaacuWGZbWCgaqbaiabg2da9iabigdaXaqaaiabdcfaqnaaBaaameaacqWFYoGyaeqaaaqdcqGHris5aaWcbaGaem4CamNaeyypa0JaeGymaedabaGaemiuaa1aaSbaaWqaaiab=f7aHbqabaaaniabggHiLdaaaOWaaabCaeaadaaeWbqaaiabd6gaUjabcIcaOiab+vha1naaBaaaleaacqWGZbWCaeqaaOGaeiykaKIaemOBa4MaeiikaGIae4xDau3aaSbaaSqaaiqbdohaZzaafaaabeaakiabcMcaPaWcbaGafm4CamNbauaacqGH9aqpcqaIXaqmaeaacqWGqbaudaWgaaadbaGae8NSdigabeaaa0GaeyyeIuoaaSqaaiabdohaZjabg2da9iabigdaXaqaaiabdcfaqnaaBaaameaacqWFXoqyaeqaaaqdcqGHris5aOWaauWaaeaacqGF1bqDdaWgaaWcbaGaem4CamhabeaakiabgkHiTiab+vha1naaBaaaleaacuWGZbWCgaqbaaqabaaakiaawMa7caGLkWoacaWLjaGaaCzcamaabmaabaGaeeyrauKaeeyCaeNaeeyDauNaeeyyaeMaeeiDaqNaeeyAaKMaee4Ba8MaeeOBa4MaeeiiaaIaeGymaedacaGLOaGaayzkaaaaaa@920C@

where *P*_*α *_and *P*_*β *_are the number of points **u**_s _and **u**_s' _used to discretize the two units or blocks *v*_*α *_and *v*_*β*_, respectively. The quantity n(**u**_s_) represents the population size within the 25 km^2 ^cell centred on the discretizing point **u**_s_. For the examples of Figure 1, the discretizing points are identified with the nodes of the 5 km grid, yielding a total of 9 to 69 points per county in Indiana, and 11 to 2,082 discretizing points for the West Coast counties. The set of block-to-block distances (Equation (1)) are plotted against Euclidian distances between polygon centroids depicted by red dots in Figure 2 (middle maps). The scatterplots at the bottom of Figure 2 indicate a high correlation between the two sets of distances; discrepancies essentially occur for neighboring counties (i.e. for small distances). The correlation is slightly smaller for Region 2 where county shapes and sizes vary greatly. Block-to-block distances are numerically very close to the distances computed between population-weighted centroids depicted by blue triangles in Figure 2 (middle maps).

### Poisson kriging: the centroid-based approach

The geostatistical methodology for the estimation of risk values from empirical frequencies, and its performance relative to common smoothers, is described in details in Goovaerts [15]. This section provides a brief recall of the centroid-based implementation of Poisson kriging (PK) for prediction of aggregated risk values. The approach is then generalized to account for spatial supports and population density in both the case of area-to-area and area-to-point predictions.

For a given number *N *of geographical units *v*_*α *_(e.g. counties), denote the observed mortality rates (areal data) as *z*(*v*_*α*_) = *d*(*v*_*α*_)/*n*(*v*_*α*_), where *d*(*v*_*α*_) is the number of recorded mortality cases and *n*(*v*_*α*_) is the size of the population at risk. Let us assume for now that all units *v*_*α *_have similar shapes and sizes, with a uniform population density. A straightforward approach to implement geostatistics is to assume that each unit or measurement support is a single point. This simplification amounts at assigning each measured rate to the geographic centroid of the unit over which it has been recorded. For example, let **u**_*α *_= (x_*α*_, y_*α*_) be the vector of spatial coordinates for the centroid of the unit *v*_*α *_. The disease count *d*(**u**_*α*_) is interpreted as a realization of a random variable *D*(**u**_*α*_) that follows a Poisson distribution with one parameter (expected number of counts) that is the product of the population size *n*(**u**_*α*_) by the local risk *R*(**u**_*α*_).

The risk over a given unit *v*_*α *_is estimated as a linear combination of the rate observed for that unit, *z*(**u**_*α*_), and in (*K*-1) neighboring units:

r^PK(uα)=∑i=1Kλi(uα)z(ui)     (Equation 2)
 MathType@MTEF@5@5@+=feaafiart1ev1aaatCvAUfKttLearuWrP9MDH5MBPbIqV92AaeXatLxBI9gBaebbnrfifHhDYfgasaacH8akY=wiFfYdH8Gipec8Eeeu0xXdbba9frFj0=OqFfea0dXdd9vqai=hGuQ8kuc9pgc9s8qqaq=dirpe0xb9q8qiLsFr0=vr0=vr0dc8meaabaqaciaacaGaaeqabaqabeGadaaakeaacuWGYbGCgaqcamaaBaaaleaacqWGqbaucqWGlbWsaeqaaOGaeiikaGccbeGae8xDau3aaSbaaSqaaGGaciab+f7aHbqabaGccqGGPaqkcqGH9aqpdaaeWbqaaiab+T7aSnaaBaaaleaacqWGPbqAaeqaaOGaeiikaGIae8xDau3aaSbaaSqaaiab+f7aHbqabaGccqGGPaqkcqWG6bGEcqGGOaakcqWF1bqDdaWgaaWcbaGaemyAaKgabeaakiabcMcaPaWcbaGaemyAaKMaeyypa0JaeGymaedabaGaem4saSeaniabggHiLdGccaWLjaGaaCzcamaabmaabaGaeeyrauKaeeyCaeNaeeyDauNaeeyyaeMaeeiDaqNaeeyAaKMaee4Ba8MaeeOBa4MaeeiiaaIaeGOmaidacaGLOaGaayzkaaaaaa@5B17@

where *λ*_*i*_(**u**_*α*_) is the weight assigned to the rate *z*(**u**_i_) when estimating the risk at **u**_*α *_. The *K *weights are the solution of the following system of linear equations:

∑j=1Kλj(uα)[CR(ui−uj)+δijm*n(ui)]+μ(uα)=CR(ui−uα)i=1,...,K∑j=1Kλj(uα)=1     (Equation 3)
 MathType@MTEF@5@5@+=feaafiart1ev1aaatCvAUfKttLearuWrP9MDH5MBPbIqV92AaeXatLxBI9gBaebbnrfifHhDYfgasaacH8akY=wiFfYdH8Gipec8Eeeu0xXdbba9frFj0=OqFfea0dXdd9vqai=hGuQ8kuc9pgc9s8qqaq=dirpe0xb9q8qiLsFr0=vr0=vr0dc8meaabaqaciaacaGaaeqabaqabeGadaaakeaafaqaaeGacaaabaWaaabCaeaaiiGacqWF7oaBdaWgaaWcbaGaemOAaOgabeaakiabcIcaOGqabiab+vha1naaBaaaleaacqWFXoqyaeqaaOGaeiykaKYaamWaaeaacqWGdbWqdaWgaaWcbaGaemOuaifabeaakiabcIcaOiab+vha1naaBaaaleaacqWGPbqAaeqaaOGaeyOeI0Iae4xDau3aaSbaaSqaaiabdQgaQbqabaGccqGGPaqkcqGHRaWkcqWF0oazdaWgaaWcbaGaemyAaKMaemOAaOgabeaakmaalaaabaGaemyBa0MaeiOkaOcabaGaemOBa4MaeiikaGIae4xDau3aaSbaaSqaaiabdMgaPbqabaGccqGGPaqkaaaacaGLBbGaayzxaaGaey4kaSIae8hVd0MaeiikaGIae4xDau3aaSbaaSqaaiab=f7aHbqabaGccqGGPaqkcqGH9aqpcqWGdbWqdaWgaaWcbaGaemOuaifabeaakiabcIcaOiab+vha1naaBaaaleaacqWGPbqAaeqaaOGaeyOeI0Iae4xDau3aaSbaaSqaaiab=f7aHbqabaGccqGGPaqkaSqaaiabdQgaQjabg2da9iabigdaXaqaaiabdUealbqdcqGHris5aaGcbaGaemyAaKMaeyypa0JaeGymaeJaeiilaWIaeiOla4IaeiOla4IaeiOla4IaeiilaWIaem4saSeabaWaaabCaeaacqWF7oaBdaWgaaWcbaGaemOAaOgabeaakiabcIcaOiab+vha1naaBaaaleaacqWFXoqyaeqaaOGaeiykaKcaleaacqWGQbGAcqGH9aqpcqaIXaqmaeaacqWGlbWsa0GaeyyeIuoakiabg2da9iabigdaXaqaaaaacaWLjaGaaCzcamaabmaabaGaeeyrauKaeeyCaeNaeeyDauNaeeyyaeMaeeiDaqNaeeyAaKMaee4Ba8MaeeOBa4MaeeiiaaIaeG4mamdacaGLOaGaayzkaaaaaa@937E@

where *δ*_*ij *_= 1 if **u**_i _= **u**_j _and 0 otherwise, and m* is the population-weighted mean of the *N *rates. The "error variance" term, m*/*n*(**u**_i_), leads to smaller weights for less reliable data (i.e. rates measured over smaller populations). The prediction variance associated with the estimate (2) is computed as:

σPK2(uα)=CR(0)−∑i=1Kλi(uα)CR(ui−uα)−μ(uα)     (Equation 4)
 MathType@MTEF@5@5@+=feaafiart1ev1aaatCvAUfKttLearuWrP9MDH5MBPbIqV92AaeXatLxBI9gBaebbnrfifHhDYfgasaacH8akY=wiFfYdH8Gipec8Eeeu0xXdbba9frFj0=OqFfea0dXdd9vqai=hGuQ8kuc9pgc9s8qqaq=dirpe0xb9q8qiLsFr0=vr0=vr0dc8meaabaqaciaacaGaaeqabaqabeGadaaakeaaiiGacqWFdpWCdaqhaaWcbaGaemiuaaLaem4saSeabaGaeGOmaidaaOGaeiikaGccbeGae4xDau3aaSbaaSqaaiab=f7aHbqabaGccqGGPaqkcqGH9aqpcqWGdbWqdaWgaaWcbaGaemOuaifabeaakiabcIcaOiabicdaWiabcMcaPiabgkHiTmaaqahabaGae83UdW2aaSbaaSqaaiabdMgaPbqabaGccqGGOaakcqGF1bqDdaWgaaWcbaGae8xSdegabeaakiabcMcaPiabdoeadnaaBaaaleaacqWGsbGuaeqaaOGaeiikaGIae4xDau3aaSbaaSqaaiabdMgaPbqabaGccqGHsislcqGF1bqDdaWgaaWcbaGae8xSdegabeaakiabcMcaPiabgkHiTiab=X7aTjabcIcaOiab+vha1naaBaaaleaacqWFXoqyaeqaaOGaeiykaKcaleaacqWGPbqAcqGH9aqpcqaIXaqmaeaacqWGlbWsa0GaeyyeIuoakiaaxMaacaWLjaWaaeWaaeaacqqGfbqrcqqGXbqCcqqG1bqDcqqGHbqycqqG0baDcqqGPbqAcqqGVbWBcqqGUbGBcqqGGaaicqaI0aanaiaawIcacaGLPaaaaaa@6EFC@

To solve the kriging system (Equation 3), one needs a model for the covariance of the risk, *C*_*R*_(**h**), or equivalently its semivariogram *γ*_*R*_(**h**) = *C*_*R*_(0)-*C*_*R*_(**h**). Following Monestiez et al. [21,22] the semivariogram of the risk is estimated as:

γ^R(h)=12∑α=1N(h)n(uα)n(uα+h)n(uα)+n(uα+h)∑α=1N(h){n(uα)n(uα+h)n(uα)+n(uα+h)[z(uα)−z(uα+h)]2−m*}     (Equation 5)
 MathType@MTEF@5@5@+=feaafiart1ev1aaatCvAUfKttLearuWrP9MDH5MBPbIqV92AaeXatLxBI9gBaebbnrfifHhDYfgasaacH8akY=wiFfYdH8Gipec8Eeeu0xXdbba9frFj0=OqFfea0dXdd9vqai=hGuQ8kuc9pgc9s8qqaq=dirpe0xb9q8qiLsFr0=vr0=vr0dc8meaabaqaciaacaGaaeqabaqabeGadaaakeaaiiGacuWFZoWzgaqcamaaBaaaleaacqWGsbGuaeqaaOGaeiikaGccbeGae4hAaGMaeiykaKIaeyypa0ZaaSaaaeaacqaIXaqmaeaacqaIYaGmdaaeWbqaamaalaaabaGaemOBa4MaeiikaGIae4xDau3aaSbaaSqaaiab=f7aHbqabaGccqGGPaqkcqWGUbGBcqGGOaakcqGF1bqDdaWgaaWcbaGae8xSdegabeaakiabgUcaRiab+HgaOjabcMcaPaqaaiabd6gaUjabcIcaOiab+vha1naaBaaaleaacqWFXoqyaeqaaOGaeiykaKIaey4kaSIaemOBa4MaeiikaGIae4xDau3aaSbaaSqaaiab=f7aHbqabaGccqGHRaWkcqGFObaAcqGGPaqkaaaaleaacqWFXoqycqGH9aqpcqaIXaqmaeaacqWGobGtcqGGOaakcqGFObaAcqGGPaqka0GaeyyeIuoaaaGcdaaeWbqaamaacmqabaWaaSaaaeaacqWGUbGBcqGGOaakcqGF1bqDdaWgaaWcbaGae8xSdegabeaakiabcMcaPiabd6gaUjabcIcaOiab+vha1naaBaaaleaacqWFXoqyaeqaaOGaey4kaSIae4hAaGMaeiykaKcabaGaemOBa4MaeiikaGIae4xDau3aaSbaaSqaaiab=f7aHbqabaGccqGGPaqkcqGHRaWkcqWGUbGBcqGGOaakcqGF1bqDdaWgaaWcbaGae8xSdegabeaakiabgUcaRiab+HgaOjabcMcaPaaadaWadaqaaiabdQha6jabcIcaOiab+vha1naaBaaaleaacqWFXoqyaeqaaOGaeiykaKIaeyOeI0IaemOEaONaeiikaGIae4xDau3aaSbaaSqaaiab=f7aHbqabaGccqGHRaWkcqGFObaAcqGGPaqkaiaawUfacaGLDbaadaahaaWcbeqaaiabikdaYaaakiabgkHiTiabd2gaTjabcQcaQaGaay5Eaiaaw2haaaWcbaGae8xSdeMaeyypa0JaeGymaedabaGaemOta4KaeiikaGIae4hAaGMaeiykaKcaniabggHiLdGccaWLjaGaaCzcamaabmaabaGaeeyrauKaeeyCaeNaeeyDauNaeeyyaeMaeeiDaqNaeeyAaKMaee4Ba8MaeeOBa4MaeeiiaaIaeGynaudacaGLOaGaayzkaaaaaa@AF0D@

The different spatial increments [*z*(**u**_*α*_)-*z*(**u**_*α*_+**h**)]^2 ^are weighted by a function of their respective population sizes, *n*(**u**_*α*_)*n*(**u**_*α *_+ **h**)/(*n*(**u**_*α*_) + *n*(**u**_*α *_+ **h**)), a term which is inversely proportional to their standard deviation. More importance is thus given to the more reliable data pairs (i.e. smaller standard deviations).

### Area-to-Area (ATA) Poisson kriging

In presence of geographical units with very different shapes and sizes, it is overly simplistic to assimilate each unit *v*_*α *_to its geographic centroid **u**_*α *_. The spatial support of each unit needs to be accounted for in both the semivariogram inference and kriging. Following the terminology in Kyriakidis [14], ATA kriging refers to the case where both the prediction and measurement supports are areas instead of points. The PK estimate (2) for the areal risk value *r*(*v*_*α*_) thus becomes:

r^PK(vα)=∑i=1Kλi(vα)z(vi)     (Equation 6)
 MathType@MTEF@5@5@+=feaafiart1ev1aaatCvAUfKttLearuWrP9MDH5MBPbIqV92AaeXatLxBI9gBaebbnrfifHhDYfgasaacH8akY=wiFfYdH8Gipec8Eeeu0xXdbba9frFj0=OqFfea0dXdd9vqai=hGuQ8kuc9pgc9s8qqaq=dirpe0xb9q8qiLsFr0=vr0=vr0dc8meaabaqaciaacaGaaeqabaqabeGadaaakeaacuWGYbGCgaqcamaaBaaaleaacqWGqbaucqWGlbWsaeqaaOGaeiikaGIaemODay3aaSbaaSqaaGGaciab=f7aHbqabaGccqGGPaqkcqGH9aqpdaaeWbqaaiab=T7aSnaaBaaaleaacqWGPbqAaeqaaOGaeiikaGIaemODay3aaSbaaSqaaiab=f7aHbqabaGccqGGPaqkcqWG6bGEcqGGOaakcqWG2bGDdaWgaaWcbaGaemyAaKgabeaakiabcMcaPaWcbaGaemyAaKMaeyypa0JaeGymaedabaGaem4saSeaniabggHiLdGccaWLjaGaaCzcamaabmaabaGaeeyrauKaeeyCaeNaeeyDauNaeeyyaeMaeeiDaqNaeeyAaKMaee4Ba8MaeeOBa4MaeeiiaaIaeGOnaydacaGLOaGaayzkaaaaaa@5B2A@

The weights *λ*_*i*_(*v*_*α*_) are computed by solving the following kriging system:

∑j=1Kλj(vα)[C¯R(vi,vj)+δijm*n(νi)]+μ(vα)=C¯R(vi,vα)i=1,...,K∑j=1Kλj(vα)=1.     (Equation 7)
 MathType@MTEF@5@5@+=feaafiart1ev1aaatCvAUfKttLearuWrP9MDH5MBPbIqV92AaeXatLxBI9gBaebbnrfifHhDYfgasaacH8akY=wiFfYdH8Gipec8Eeeu0xXdbba9frFj0=OqFfea0dXdd9vqai=hGuQ8kuc9pgc9s8qqaq=dirpe0xb9q8qiLsFr0=vr0=vr0dc8meaabaqaciaacaGaaeqabaqabeGadaaakeaafaqaaeGacaaabaWaaabCaeaaiiGacqWF7oaBdaWgaaWcbaGaemOAaOgabeaakiabcIcaOiabdAha2naaBaaaleaacqWFXoqyaeqaaOGaeiykaKYaamWaaeaacuWGdbWqgaqeamaaBaaaleaacqWGsbGuaeqaaOGaeiikaGIaemODay3aaSbaaSqaaiabdMgaPbqabaGccqGGSaalcqWG2bGDdaWgaaWcbaGaemOAaOgabeaakiabcMcaPiabgUcaRiab=r7aKnaaBaaaleaacqWGPbqAcqWGQbGAaeqaaOWaaSaaaeaacqWGTbqBcqGGQaGkaeaacqWGUbGBcqGGOaakcqWF9oGBdaWgaaWcbaGaemyAaKgabeaakiabcMcaPaaaaiaawUfacaGLDbaaaSqaaiabdQgaQjabg2da9iabigdaXaqaaiabdUealbqdcqGHris5aOGaey4kaSIae8hVd0MaeiikaGIaemODay3aaSbaaSqaaiab=f7aHbqabaGccqGGPaqkcqGH9aqpcuWGdbWqgaqeamaaBaaaleaacqWGsbGuaeqaaOGaeiikaGIaemODay3aaSbaaSqaaiabdMgaPbqabaGccqGGSaalcqWG2bGDdaWgaaWcbaGae8xSdegabeaakiabcMcaPaqaaiabbMgaPjabg2da9iabigdaXiabcYcaSiabc6caUiabc6caUiabc6caUiabcYcaSiabdUealbqaamaaqahabaGae83UdW2aaSbaaSqaaiabdQgaQbqabaGccqGGOaakcqWG2bGDdaWgaaWcbaGae8xSdegabeaakiabcMcaPiabg2da9iabigdaXiabc6caUaWcbaGaemOAaOMaeyypa0JaeGymaedabaGaem4saSeaniabggHiLdaakeaaaaGaaCzcaiaaxMaadaqadaqaaiabbweafjabbghaXjabbwha1jabbggaHjabbsha0jabbMgaPjabb+gaVjabb6gaUjabbccaGiabiEda3aGaayjkaiaawMcaaaaa@94EA@

The main difference between systems (3) and (7) is that point-to-point covariance terms *C*_*R*_(**u**_*i *_- **u**_*j*_) are replaced by area-to-area covariances C¯
 MathType@MTEF@5@5@+=feaafiart1ev1aaatCvAUfKttLearuWrP9MDH5MBPbIqV92AaeXatLxBI9gBaebbnrfifHhDYfgasaacH8akY=wiFfYdH8Gipec8Eeeu0xXdbba9frFj0=OqFfea0dXdd9vqai=hGuQ8kuc9pgc9s8qqaq=dirpe0xb9q8qiLsFr0=vr0=vr0dc8meaabaqaciaacaGaaeqabaqabeGadaaakeaacuWGdbWqgaqeaaaa@2DD3@_*R*_(*v*_*i*_, *v*_*j*_) = Cov{*Z*(*v*_i_), *Z*(*v*_j_)}. Those covariances are numerically approximated by averaging the point-support covariance *C*(**h**) computed between any two locations discretizing the areas *v*_i _and *v*_j_:

C¯R(vi,vj)=1∑s=1Pi∑s′=1Pjwss′∑s=1Pi∑s′=1Pjwss′C(us,us′)     (Equation 8)
 MathType@MTEF@5@5@+=feaafiart1ev1aaatCvAUfKttLearuWrP9MDH5MBPbIqV92AaeXatLxBI9gBaebbnrfifHhDYfgasaacH8akY=wiFfYdH8Gipec8Eeeu0xXdbba9frFj0=OqFfea0dXdd9vqai=hGuQ8kuc9pgc9s8qqaq=dirpe0xb9q8qiLsFr0=vr0=vr0dc8meaabaqaciaacaGaaeqabaqabeGadaaakeaacuWGdbWqgaqeamaaBaaaleaacqWGsbGuaeqaaOGaeiikaGIaemODay3aaSbaaSqaaiabdMgaPbqabaGccqGGSaalcqWG2bGDdaWgaaWcbaGaemOAaOgabeaakiabcMcaPiabg2da9maalaaabaGaeGymaedabaWaaabCaeaadaaeWbqaaiabdEha3naaBaaaleaacqWGZbWCcuWGZbWCgaqbaaqabaaabaGafm4CamNbauaacqGH9aqpcqaIXaqmaeaacqWGqbaudaWgaaadbaGaemOAaOgabeaaa0GaeyyeIuoaaSqaaiabdohaZjabg2da9iabigdaXaqaaiabdcfaqnaaBaaameaacqWGPbqAaeqaaaqdcqGHris5aaaakmaaqahabaWaaabCaeaacqWG3bWDdaWgaaWcbaGaem4CamNafm4CamNbauaaaeqaaaqaaiqbdohaZzaafaGaeyypa0JaeGymaedabaGaemiuaa1aaSbaaWqaaiabdQgaQbqabaaaniabggHiLdaaleaacqWGZbWCcqGH9aqpcqaIXaqmaeaacqWGqbaudaWgaaadbaGaemyAaKgabeaaa0GaeyyeIuoakiabdoeadjabcIcaOGqabiab=vha1naaBaaaleaacqWGZbWCaeqaaOGaeiilaWIae8xDau3aaSbaaSqaaiqbdohaZzaafaaabeaakiabcMcaPiaaxMaacaWLjaWaaeWaaeaacqqGfbqrcqqGXbqCcqqG1bqDcqqGHbqycqqG0baDcqqGPbqAcqqGVbWBcqqGUbGBcqqGGaaicqaI4aaoaiaawIcacaGLPaaaaaa@7DA5@

where *P*_*i *_and *P*_*j *_are the number of points used to discretize the two areas *v*_i _and *v*_j_, respectively. The weights *w*_*ss*' _are computed as the product of population sizes within the square cells centred on the discretizing point **u**_s _and **u**_s'_:

wss′=n(us)×n(us′) with ∑s=1Pin(us)=n(vi) and ∑s′=1Pjn(us′)=n(vj)     (Equation 9)
 MathType@MTEF@5@5@+=feaafiart1ev1aaatCvAUfKttLearuWrP9MDH5MBPbIqV92AaeXatLxBI9gBaebbnrfifHhDYfgasaacH8akY=wiFfYdH8Gipec8Eeeu0xXdbba9frFj0=OqFfea0dXdd9vqai=hGuQ8kuc9pgc9s8qqaq=dirpe0xb9q8qiLsFr0=vr0=vr0dc8meaabaqaciaacaGaaeqabaqabeGadaaakeaacqWG3bWDdaWgaaWcbaGaem4CamNafm4CamNbauaaaeqaaOGaeyypa0JaemOBa4MaeiikaGccbeGae8xDau3aaSbaaSqaaiabdohaZbqabaGccqGGPaqkcqGHxdaTcqWGUbGBcqGGOaakcqWF1bqDdaWgaaWcbaGafm4CamNbauaaaeqaaOGaeiykaKIaeeiiaaIaee4DaCNaeeyAaKMaeeiDaqNaeeiAaGMaeeiiaaYaaabCaeaacqWGUbGBcqGGOaakcqWF1bqDdaWgaaWcbaGaem4CamhabeaakiabcMcaPiabg2da9iabd6gaUjabcIcaOiabdAha2naaBaaaleaacqWGPbqAaeqaaOGaeiykaKcaleaacqWGZbWCcqGH9aqpcqaIXaqmaeaacqWGqbaudaWgaaadbaGaemyAaKgabeaaa0GaeyyeIuoakiabbccaGiabbggaHjabb6gaUjabbsgaKjabbccaGmaaqahabaGaemOBa4MaeiikaGIae8xDau3aaSbaaSqaaiqbdohaZzaafaaabeaakiabcMcaPiabg2da9iabd6gaUjabcIcaOiabdAha2naaBaaaleaacqWGQbGAaeqaaOGaeiykaKcaleaacuWGZbWCgaqbaiabg2da9iabigdaXaqaaiabdcfaqnaaBaaameaacqWGQbGAaeqaaaqdcqGHris5aOGaaCzcaiaaxMaadaqadaqaaiabbweafjabbghaXjabbwha1jabbggaHjabbsha0jabbMgaPjabb+gaVjabb6gaUjabbccaGiabiMda5aGaayjkaiaawMcaaaaa@884B@

The kriging variance for the ATA kriging estimator is computed as:

σPK2(vα)=C¯R(vα,vα)−∑i=1Kλi(vα)C¯R(vi,vα)−μ(vα)     (Equation 10)
 MathType@MTEF@5@5@+=feaafiart1ev1aaatCvAUfKttLearuWrP9MDH5MBPbIqV92AaeXatLxBI9gBaebbnrfifHhDYfgasaacH8akY=wiFfYdH8Gipec8Eeeu0xXdbba9frFj0=OqFfea0dXdd9vqai=hGuQ8kuc9pgc9s8qqaq=dirpe0xb9q8qiLsFr0=vr0=vr0dc8meaabaqaciaacaGaaeqabaqabeGadaaakeaaiiGacqWFdpWCdaqhaaWcbaGaemiuaaLaem4saSeabaGaeGOmaidaaOGaeiikaGIaemODay3aaSbaaSqaaiab=f7aHbqabaGccqGGPaqkcqGH9aqpcuWGdbWqgaqeamaaBaaaleaacqWGsbGuaeqaaOGaeiikaGIaemODay3aaSbaaSqaaiab=f7aHbqabaGccqGGSaalcqWG2bGDdaWgaaWcbaGae8xSdegabeaakiabcMcaPiabgkHiTmaaqahabaGae83UdW2aaSbaaSqaaiabdMgaPbqabaaabaGaemyAaKMaeyypa0JaeGymaedabaGaem4saSeaniabggHiLdGccqGGOaakcqWG2bGDdaWgaaWcbaGae8xSdegabeaakiabcMcaPiqbdoeadzaaraWaaSbaaSqaaiabdkfasbqabaGccqGGOaakcqWG2bGDdaWgaaWcbaGaemyAaKgabeaakiabcYcaSiabdAha2naaBaaaleaacqWFXoqyaeqaaOGaeiykaKIaeyOeI0Iae8hVd0MaeiikaGIaemODay3aaSbaaSqaaiab=f7aHbqabaGccqGGPaqkcaWLjaGaaCzcamaabmaabaGaeeyrauKaeeyCaeNaeeyDauNaeeyyaeMaeeiDaqNaeeyAaKMaee4Ba8MaeeOBa4MaeeiiaaIaeGymaeJaeGimaadacaGLOaGaayzkaaaaaa@7687@

where C¯
 MathType@MTEF@5@5@+=feaafiart1ev1aaatCvAUfKttLearuWrP9MDH5MBPbIqV92AaeXatLxBI9gBaebbnrfifHhDYfgasaacH8akY=wiFfYdH8Gipec8Eeeu0xXdbba9frFj0=OqFfea0dXdd9vqai=hGuQ8kuc9pgc9s8qqaq=dirpe0xb9q8qiLsFr0=vr0=vr0dc8meaabaqaciaacaGaaeqabaqabeGadaaakeaacuWGdbWqgaqeaaaa@2DD3@_*R*_(*v*_*α*_, *v*_*α*_) is the within-area covariance that is computed according to Equation (8) with *v*_i _= *v*_j _= *v*_*α*_.

### Area-to-Point (ATP) Poisson kriging

A particular case of ATA kriging is when the prediction support is so small that it can be assimilated to a point **u**_s_, leading to the following area-to-point kriging estimator and kriging variance:

r^PK(us)=∑i=1Kλi(us)z(vi)     (Equation 11)
 MathType@MTEF@5@5@+=feaafiart1ev1aaatCvAUfKttLearuWrP9MDH5MBPbIqV92AaeXatLxBI9gBaebbnrfifHhDYfgasaacH8akY=wiFfYdH8Gipec8Eeeu0xXdbba9frFj0=OqFfea0dXdd9vqai=hGuQ8kuc9pgc9s8qqaq=dirpe0xb9q8qiLsFr0=vr0=vr0dc8meaabaqaciaacaGaaeqabaqabeGadaaakeaacuWGYbGCgaqcamaaBaaaleaacqWGqbaucqWGlbWsaeqaaOGaeiikaGccbeGae8xDau3aaSbaaSqaaiabdohaZbqabaGccqGGPaqkcqGH9aqpdaaeWbqaaGGaciab+T7aSnaaBaaaleaacqWGPbqAaeqaaOGaeiikaGIae8xDau3aaSbaaSqaaiabdohaZbqabaGccqGGPaqkcqWG6bGEcqGGOaakcqWG2bGDdaWgaaWcbaGaemyAaKgabeaakiabcMcaPaWcbaGaemyAaKMaeyypa0JaeGymaedabaGaem4saSeaniabggHiLdGccaWLjaGaaCzcamaabmaabaGaeeyrauKaeeyCaeNaeeyDauNaeeyyaeMaeeiDaqNaeeyAaKMaee4Ba8MaeeOBa4MaeeiiaaIaeGymaeJaeGymaedacaGLOaGaayzkaaaaaa@5BB7@

σPK2(us)=CR(0)−∑i=1Kλi(us)C¯R(vi,us)−μ(us)     (Equation 12)
 MathType@MTEF@5@5@+=feaafiart1ev1aaatCvAUfKttLearuWrP9MDH5MBPbIqV92AaeXatLxBI9gBaebbnrfifHhDYfgasaacH8akY=wiFfYdH8Gipec8Eeeu0xXdbba9frFj0=OqFfea0dXdd9vqai=hGuQ8kuc9pgc9s8qqaq=dirpe0xb9q8qiLsFr0=vr0=vr0dc8meaabaqaciaacaGaaeqabaqabeGadaaakeaaiiGacqWFdpWCdaqhaaWcbaGaemiuaaLaem4saSeabaGaeGOmaidaaOGaeiikaGccbeGae4xDau3aaSbaaSqaaiabdohaZbqabaGccqGGPaqkcqGH9aqpcqWGdbWqdaWgaaWcbaGaemOuaifabeaakiabcIcaOiabicdaWiabcMcaPiabgkHiTmaaqahabaGae83UdW2aaSbaaSqaaiabdMgaPbqabaaabaGaemyAaKMaeyypa0JaeGymaedabaGaem4saSeaniabggHiLdGccqGGOaakcqGF1bqDdaWgaaWcbaGaem4CamhabeaakiabcMcaPiqbdoeadzaaraWaaSbaaSqaaiabdkfasbqabaGccqGGOaakcqWG2bGDdaWgaaWcbaGaemyAaKgabeaakiabcYcaSiab+vha1naaBaaaleaacqWGZbWCaeqaaOGaeiykaKIaeyOeI0Iae8hVd0MaeiikaGIae4xDau3aaSbaaSqaaiabdohaZbqabaGccqGGPaqkcaWLjaGaaCzcamaabmaabaGaeeyrauKaeeyCaeNaeeyDauNaeeyyaeMaeeiDaqNaeeyAaKMaee4Ba8MaeeOBa4MaeeiiaaIaeGymaeJaeGOmaidacaGLOaGaayzkaaaaaa@6F39@

The kriging weights and the Lagrange parameter *μ*(**u**_s_) are computed by solving the following system of linear equations:

∑j=1Kλj(us)[C¯R(vi,vj)+δijm∗n(vi)]+μ(us)=C¯R(vi,us)i=1,...,K∑j=1Kλj(us)=1.     (Equation 13)
 MathType@MTEF@5@5@+=feaafiart1ev1aaatCvAUfKttLearuWrP9MDH5MBPbIqV92AaeXatLxBI9gBaebbnrfifHhDYfgasaacH8akY=wiFfYdH8Gipec8Eeeu0xXdbba9frFj0=OqFfea0dXdd9vqai=hGuQ8kuc9pgc9s8qqaq=dirpe0xb9q8qiLsFr0=vr0=vr0dc8meaabaqaciaacaGaaeqabaqabeGadaaakeaafaqaaeGacaaabaWaaabCaeaaiiGacqWF7oaBdaWgaaWcbaGaemOAaOgabeaaaeaacqWGQbGAcqGH9aqpcqaIXaqmaeaacqWGlbWsa0GaeyyeIuoakiabcIcaOGqabiab+vha1naaBaaaleaacqWGZbWCaeqaaOGaeiykaKYaamWaaeaacuWGdbWqgaqeamaaBaaaleaacqWGsbGuaeqaaOGaeiikaGIaemODay3aaSbaaSqaaiabdMgaPbqabaGccqGGSaalcqWG2bGDdaWgaaWcbaGaemOAaOgabeaakiabcMcaPiabgUcaRiab=r7aKnaaBaaaleaacqWGPbqAcqWGQbGAaeqaaOWaaSaaaeaacqWGTbqBcqGHxiIkaeaacqWGUbGBcqGGOaakcqWG2bGDdaWgaaWcbaGaemyAaKgabeaakiabcMcaPaaaaiaawUfacaGLDbaacqGHRaWkcqWF8oqBcqGGOaakcqGF1bqDdaWgaaWcbaGaem4CamhabeaakiabcMcaPiabg2da9iqbdoeadzaaraWaaSbaaSqaaiabdkfasbqabaGccqGGOaakcqWG2bGDdaWgaaWcbaGaemyAaKgabeaaieaakiab9XcaSiab+vha1naaBaaaleaacqWGZbWCaeqaaOGaeiykaKcabaGaeeyAaKMaeyypa0JaeGymaeJaeiilaWIaeiOla4IaeiOla4IaeiOla4IaeiilaWIaem4saSeabaWaaabCaeaacqWF7oaBdaWgaaWcbaGaemOAaOgabeaakiabcIcaOiab+vha1naaBaaaleaacqWGZbWCaeqaaOGaeiykaKIaeyypa0JaeGymaeJaeiOla4caleaacqWGQbGAcqGH9aqpcqaIXaqmaeaacqWGlbWsa0GaeyyeIuoaaOqaaaaacaWLjaGaaCzcamaabmaabaGaeeyrauKaeeyCaeNaeeyDauNaeeyyaeMaeeiDaqNaeeyAaKMaee4Ba8MaeeOBa4MaeeiiaaIaeGymaeJaeG4mamdacaGLOaGaayzkaaaaaa@94D8@

The ATP kriging system is similar to the ATA kriging system (Equation 7), except for the right-hand-side term where the area-to-area covariances C¯
 MathType@MTEF@5@5@+=feaafiart1ev1aaatCvAUfKttLearuWrP9MDH5MBPbIqV92AaeXatLxBI9gBaebbnrfifHhDYfgasaacH8akY=wiFfYdH8Gipec8Eeeu0xXdbba9frFj0=OqFfea0dXdd9vqai=hGuQ8kuc9pgc9s8qqaq=dirpe0xb9q8qiLsFr0=vr0=vr0dc8meaabaqaciaacaGaaeqabaqabeGadaaakeaacuWGdbWqgaqeaaaa@2DD3@_*R*_(*v*_*i*_, *v*_*α*_) are replaced by area-to-point covariances C¯
 MathType@MTEF@5@5@+=feaafiart1ev1aaatCvAUfKttLearuWrP9MDH5MBPbIqV92AaeXatLxBI9gBaebbnrfifHhDYfgasaacH8akY=wiFfYdH8Gipec8Eeeu0xXdbba9frFj0=OqFfea0dXdd9vqai=hGuQ8kuc9pgc9s8qqaq=dirpe0xb9q8qiLsFr0=vr0=vr0dc8meaabaqaciaacaGaaeqabaqabeGadaaakeaacuWGdbWqgaqeaaaa@2DD3@_*R *_(*v*_*i*_, **u**_*s*_) that are approximated as:

C¯R(vi,us)=1∑s′=1Piws′s∑s′=1Piws′sC(us′,us)     (Equation 14)
 MathType@MTEF@5@5@+=feaafiart1ev1aaatCvAUfKttLearuWrP9MDH5MBPbIqV92AaeXatLxBI9gBaebbnrfifHhDYfgasaacH8akY=wiFfYdH8Gipec8Eeeu0xXdbba9frFj0=OqFfea0dXdd9vqai=hGuQ8kuc9pgc9s8qqaq=dirpe0xb9q8qiLsFr0=vr0=vr0dc8meaabaqaciaacaGaaeqabaqabeGadaaakeaacuWGdbWqgaqeamaaBaaaleaacqWGsbGuaeqaaOGaeiikaGIaemODay3aaSbaaSqaaiabdMgaPbqabaGccqGGSaalieqacqWF1bqDdaWgaaWcbaGaem4CamhabeaakiabcMcaPiabg2da9maalaaabaGaeGymaedabaWaaabCaeaacqWG3bWDdaWgaaWcbaGafm4CamNbauaacqWGZbWCaeqaaaqaaiqbdohaZzaafaGaeyypa0JaeGymaedabaGaemiuaa1aaSbaaWqaaiabdMgaPbqabaaaniabggHiLdaaaOWaaabCaeaacqWG3bWDdaWgaaWcbaGafm4CamNbauaacqWGZbWCaeqaaOGaem4qamKaeiikaGIae8xDau3aaSbaaSqaaiqbdohaZzaafaaabeaakiabcYcaSiab=vha1naaBaaaleaacqWGZbWCaeqaaOGaeiykaKcaleaacuWGZbWCgaqbaiabg2da9iabigdaXaqaaiabdcfaqnaaBaaameaacqWGPbqAaeqaaaqdcqGHris5aOGaaCzcaiaaxMaadaqadaqaaiabbweafjabbghaXjabbwha1jabbggaHjabbsha0jabbMgaPjabb+gaVjabb6gaUjabbccaGiabigdaXiabisda0aGaayjkaiaawMcaaaaa@6DFA@

where *P*_*i *_is the number of points used to discretize the area *v*_i _and the weights *w*_*s*'*s *_are computed according to expression (9).

ATP kriging can be conducted at each node of a grid covering the study area, resulting in a continuous (isopleth) map of mortality risk and reducing the visual bias that is typically associated with the interpretation of choropleth maps. Another interesting property of the ATP kriging estimator is its coherence: the population-weighted average of the risk values estimated at the *P*_*α *_points **u**_s _discretizing a given entity *v*_*α *_yields the ATA risk estimate for this entity:

r^PK(vα)=1n(vα)∑s=1Pαn(us)r^PK(us)     (Equation 15)
 MathType@MTEF@5@5@+=feaafiart1ev1aaatCvAUfKttLearuWrP9MDH5MBPbIqV92AaeXatLxBI9gBaebbnrfifHhDYfgasaacH8akY=wiFfYdH8Gipec8Eeeu0xXdbba9frFj0=OqFfea0dXdd9vqai=hGuQ8kuc9pgc9s8qqaq=dirpe0xb9q8qiLsFr0=vr0=vr0dc8meaabaqaciaacaGaaeqabaqabeGadaaakeaacuWGYbGCgaqcamaaBaaaleaacqWGqbaucqWGlbWsaeqaaOGaeiikaGIaemODay3aaSbaaSqaaGGaciab=f7aHbqabaGccqGGPaqkcqGH9aqpdaWcaaqaaiabigdaXaqaaiabd6gaUjabcIcaOiabdAha2naaBaaaleaacqWFXoqyaeqaaOGaeiykaKcaamaaqahabaGaemOBa4MaeiikaGccbeGae4xDau3aaSbaaSqaaiabdohaZbqabaGccqGGPaqkcuWGYbGCgaqcamaaBaaaleaacqWGqbaucqWGlbWsaeqaaOGaeiikaGIae4xDau3aaSbaaSqaaiabdohaZbqabaGccqGGPaqkaSqaaiabdohaZjabg2da9iabigdaXaqaaiabdcfaqnaaBaaameaacqWFXoqyaeqaaaqdcqGHris5aOGaaCzcaiaaxMaadaqadaqaaiabbweafjabbghaXjabbwha1jabbggaHjabbsha0jabbMgaPjabb+gaVjabb6gaUjabbccaGiabigdaXiabiwda1aGaayjkaiaawMcaaaaa@65E1@

Constraint (15) is satisfied if the same *K *areal data are used for the ATP kriging of the P_*α *_risk values. Indeed, in this case the population-weighted average of the right-hand-side covariance terms of the *K *ATP kriging systems is equal to the right-hand-side covariance of the single ATA kriging system:

1n(vα)∑s=1Pαn(us)C¯R(vi,us)=1n(vα)∑s=1Pαn(us)[1n(vi)∑s′=1Pin(us′)C(us′,us)]=C¯R(vi,vα),     (Equation 16)
 MathType@MTEF@5@5@+=feaafiart1ev1aaatCvAUfKttLearuWrP9MDH5MBPbIqV92AaeXatLxBI9gBaebbnrfifHhDYfgasaacH8akY=wiFfYdH8Gipec8Eeeu0xXdbba9frFj0=OqFfea0dXdd9vqai=hGuQ8kuc9pgc9s8qqaq=dirpe0xb9q8qiLsFr0=vr0=vr0dc8meaabaqaciaacaGaaeqabaqabeGadaaakeaafaqabeGabaaabaWaaSaaaeaacqaIXaqmaeaacqWGUbGBcqGGOaakcqWG2bGDdaWgaaWcbaacciGae8xSdegabeaakiabcMcaPaaadaaeWbqaaiabd6gaUjabcIcaOGqabiab+vha1naaBaaaleaacqWGZbWCaeqaaOGaeiykaKIafm4qamKbaebadaWgaaWcbaGaemOuaifabeaaaeaacqWGZbWCcqGH9aqpcqaIXaqmaeaacqWGqbaudaWgaaadbaGae8xSdegabeaaa0GaeyyeIuoakiabcIcaOiabdAha2naaBaaaleaacqWGPbqAaeqaaOGaeiilaWIae4xDau3aaSbaaSqaaiabdohaZbqabaGccqGGPaqkcqGH9aqpdaWcaaqaaiabigdaXaqaaiabd6gaUjabcIcaOiabdAha2naaBaaaleaacqWFXoqyaeqaaOGaeiykaKcaamaaqahabaGaemOBa4MaeiikaGIae4xDau3aaSbaaSqaaiabdohaZbqabaGccqGGPaqkaSqaaiabdohaZjabg2da9iabigdaXaqaaiabdcfaqnaaBaaameaacqWFXoqyaeqaaaqdcqGHris5aOWaamWaaeaadaWcaaqaaiabigdaXaqaaiabd6gaUjabcIcaOiabdAha2naaBaaaleaacqWGPbqAaeqaaOGaeiykaKcaamaaqahabaGaemOBa4MaeiikaGIae4xDau3aaSbaaSqaaiqbdohaZzaafaaabeaakiabcMcaPiabdoeadjabcIcaOiab+vha1naaBaaaleaacuWGZbWCgaqbaaqabaGccqGGSaalcqGF1bqDdaWgaaWcbaGaem4CamhabeaakiabcMcaPaWcbaGafm4CamNbauaacqGH9aqpcqaIXaqmaeaacqWGqbaudaWgaaadbaGaemyAaKgabeaaa0GaeyyeIuoaaOGaay5waiaaw2faaaqaaiabg2da9iqbdoeadzaaraWaaSbaaSqaaiabdkfasbqabaGccqGGOaakcqWG2bGDdaWgaaWcbaGaemyAaKgabeaakiabcYcaSiabdAha2naaBaaaleaacqWFXoqyaeqaaOGaeiykaKIaeiilaWcaaiaaxMaacaWLjaWaaeWaaeaacqqGfbqrcqqGXbqCcqqG1bqDcqqGHbqycqqG0baDcqqGPbqAcqqGVbWBcqqGUbGBcqqGGaaicqaIXaqmcqaI2aGnaiaawIcacaGLPaaaaaa@A458@

per relations (8), (9) and (14). Therefore, the following relationship exists between the two sets of ATA and ATP kriging weights:

λi(vα)=1n(vα)∑s=1Pαn(us)λi(us)i=1,...,K     (Equation 17)
 MathType@MTEF@5@5@+=feaafiart1ev1aaatCvAUfKttLearuWrP9MDH5MBPbIqV92AaeXatLxBI9gBaebbnrfifHhDYfgasaacH8akY=wiFfYdH8Gipec8Eeeu0xXdbba9frFj0=OqFfea0dXdd9vqai=hGuQ8kuc9pgc9s8qqaq=dirpe0xb9q8qiLsFr0=vr0=vr0dc8meaabaqaciaacaGaaeqabaqabeGadaaakeaafaqabeqacaaabaacciGae83UdW2aaSbaaSqaaiabdMgaPbqabaGccqGGOaakcqWG2bGDdaWgaaWcbaGae8xSdegabeaakiabcMcaPiabg2da9maalaaabaGaeGymaedabaGaemOBa4MaeiikaGIaemODay3aaSbaaSqaaiab=f7aHbqabaGccqGGPaqkaaWaaabCaeaacqWGUbGBcqGGOaakieqacqGF1bqDdaWgaaWcbaGaem4CamhabeaakiabcMcaPiabeU7aSnaaBaaaleaacqWGPbqAaeqaaaqaaiabdohaZjabg2da9iabigdaXaqaaiabdcfaqnaaBaaameaacqWFXoqyaeqaaaqdcqGHris5aOGaeiikaGIae4xDau3aaSbaaSqaaiabdohaZbqabaGccqGGPaqkaeaacqWGPbqAcqGH9aqpcqaIXaqmcqGGSaalcqGGUaGlcqGGUaGlcqGGUaGlcqGGSaalcqWGlbWsaaGaaCzcaiaaxMaadaqadaqaaiabbweafjabbghaXjabbwha1jabbggaHjabbsha0jabbMgaPjabb+gaVjabb6gaUjabbccaGiabigdaXiabiEda3aGaayjkaiaawMcaaaaa@6D48@

### Deconvolution of the semivariogram of the risk

To solve both ATA and ATP kriging systems, one needs to know the point-support covariance of the risk *C*(**h**), or equivalently the point-support semivariogram *γ*(**h**). This function cannot be estimated directly from the observed rates, since only aggregated data are available. Derivation of a point-support semivariogram from the "regularized" experimental semivariogram computed from areal data is called "deconvolution" [14,28]. Unlike the mining applications that have led to the initial development of geostatistical deconvolution [8], the size and shape of geographical units in health science applications can vary greatly, which makes the deconvolution much more challenging. Goovaerts [10] developed an innovative approach to conduct the deconvolution in presence of irregular units and heterogeneous population distributions. Like most inverse problems, the deconvolution is best tackled using an iterative procedure. The basic idea is to seek the point-support model that, once regularized, is the closest to the model fitted to areal data. Only the most salient features of the deconvolution procedure are described hereafter, and the reader is referred to Goovaerts [10] for a detailed presentation of the algorithm and demonstration of its performances in simulation studies.

The deconvolution is based on the following relationship between the regularized semivariogram model *γ*_*v*_*(***h***) *fitted to areal data and area-to-area semivariogram values that are a function of the unknown point-support model *γ*(**h**):

*γ*_*v*_(*h*) = γ¯
 MathType@MTEF@5@5@+=feaafiart1ev1aaatCvAUfKttLearuWrP9MDH5MBPbIqV92AaeXatLxBI9gBaebbnrfifHhDYfgasaacH8akY=wiFfYdH8Gipec8Eeeu0xXdbba9frFj0=OqFfea0dXdd9vqai=hGuQ8kuc9pgc9s8qqaq=dirpe0xb9q8qiLsFr0=vr0=vr0dc8meaabaqaciaacaGaaeqabaqabeGadaaakeaaiiGacuWFZoWzgaqeaaaa@2E72@(*v*, *v*_*h*_) - γ¯
 MathType@MTEF@5@5@+=feaafiart1ev1aaatCvAUfKttLearuWrP9MDH5MBPbIqV92AaeXatLxBI9gBaebbnrfifHhDYfgasaacH8akY=wiFfYdH8Gipec8Eeeu0xXdbba9frFj0=OqFfea0dXdd9vqai=hGuQ8kuc9pgc9s8qqaq=dirpe0xb9q8qiLsFr0=vr0=vr0dc8meaabaqaciaacaGaaeqabaqabeGadaaakeaaiiGacuWFZoWzgaqeaaaa@2E72@_*h*_(*v*, *v*)     (Equation 18)

The area-to-area semivariogram value γ¯
 MathType@MTEF@5@5@+=feaafiart1ev1aaatCvAUfKttLearuWrP9MDH5MBPbIqV92AaeXatLxBI9gBaebbnrfifHhDYfgasaacH8akY=wiFfYdH8Gipec8Eeeu0xXdbba9frFj0=OqFfea0dXdd9vqai=hGuQ8kuc9pgc9s8qqaq=dirpe0xb9q8qiLsFr0=vr0=vr0dc8meaabaqaciaacaGaaeqabaqabeGadaaakeaaiiGacuWFZoWzgaqeaaaa@2E72@(*v*, *v*_*h*_) represents the mean value of the point-support semivariogram between an arbitrary point in the support *v *and another in the translated support *v*_h_. Because each area (e.g. county) has potentially a different size and shape, one needs to consider any two areas that can be encountered across the study area and average the semivariogram values over all N(h) pairs of areas separated by the distance *h*. More precisely, the area-to-area semivariogram value is estimated as:

γ¯(v,vh)=1N(h)∑α=1N(h)γ¯(vα,vα+h)     (Equation 19)
 MathType@MTEF@5@5@+=feaafiart1ev1aaatCvAUfKttLearuWrP9MDH5MBPbIqV92AaeXatLxBI9gBaebbnrfifHhDYfgasaacH8akY=wiFfYdH8Gipec8Eeeu0xXdbba9frFj0=OqFfea0dXdd9vqai=hGuQ8kuc9pgc9s8qqaq=dirpe0xb9q8qiLsFr0=vr0=vr0dc8meaabaqaciaacaGaaeqabaqabeGadaaakeaaiiGacuWFZoWzgaqeaiabcIcaOiabdAha2jabcYcaSiabdAha2naaBaaaleaacqWGObaAaeqaaOGaeiykaKIaeyypa0ZaaSaaaeaacqaIXaqmaeaacqWGobGtcqGGOaakcqWGObaAcqGGPaqkaaWaaabCaeaacuWFZoWzgaqeaiabcIcaOiabdAha2naaBaaaleaacqWFXoqyaeqaaOGaeiilaWIaemODay3aaSbaaSqaaiab=f7aHjabgUcaRiabdIgaObqabaGccqGGPaqkaSqaaiab=f7aHjabg2da9iabigdaXaqaaiabd6eaojabcIcaOiabdIgaOjabcMcaPaqdcqGHris5aOGaaCzcaiaaxMaadaqadaqaaiabbweafjabbghaXjabbwha1jabbggaHjabbsha0jabbMgaPjabb+gaVjabb6gaUjabbccaGiabigdaXiabiMda5aGaayjkaiaawMcaaaaa@6318@

The semivariogram value between any two areas, *v*_*α *_and *v*_*α*+*h*_, separated by the population-based distance *h *(Equation 1) is computed as:

γ¯(vα,vα+h)=1PαPα+h∑s=1Pα∑s′=1Pα+hγ(us,us′)     (Equation 20)
 MathType@MTEF@5@5@+=feaafiart1ev1aaatCvAUfKttLearuWrP9MDH5MBPbIqV92AaeXatLxBI9gBaebbnrfifHhDYfgasaacH8akY=wiFfYdH8Gipec8Eeeu0xXdbba9frFj0=OqFfea0dXdd9vqai=hGuQ8kuc9pgc9s8qqaq=dirpe0xb9q8qiLsFr0=vr0=vr0dc8meaabaqaciaacaGaaeqabaqabeGadaaakeaaiiGacuWFZoWzgaqeaiabcIcaOiabdAha2naaBaaaleaacqWFXoqyaeqaaOGaeiilaWIaemODay3aaSbaaSqaaiab=f7aHjabgUcaRiabdIgaObqabaGccqGGPaqkcqGH9aqpdaWcaaqaaiabigdaXaqaaiabdcfaqnaaBaaaleaacqWFXoqyaeqaaOGaemiuaa1aaSbaaSqaaiab=f7aHjabgUcaRiabdIgaObqabaaaaOWaaabCaeaadaaeWbqaaiab=n7aNbWcbaGafm4CamNbauaacqGH9aqpcqaIXaqmaeaacqWGqbaudaWgaaadbaGae8xSdeMaey4kaSIaemiAaGgabeaaa0GaeyyeIuoaaSqaaiabdohaZjabg2da9iabigdaXaqaaiabdcfaqnaaBaaameaacqWFXoqyaeqaaaqdcqGHris5aOGaeiikaGccbeGae4xDau3aaSbaaSqaaiabdohaZbqabaGccqGGSaalcqGF1bqDdaWgaaWcbaGafm4CamNbauaaaeqaaOGaeiykaKIaaCzcaiaaxMaadaqadaqaaiabbweafjabbghaXjabbwha1jabbggaHjabbsha0jabbMgaPjabb+gaVjabb6gaUjabbccaGiabikdaYiabicdaWaGaayjkaiaawMcaaaaa@7223@

where *P*_*α *_and *P*_*α*+*h *_are the number of points used to discretize the two areas *v*_*α *_and *v*_*α*+*h*_, respectively.

The second term in the regularization expression (Equation 18) is the within-area semivariogram value γ¯
 MathType@MTEF@5@5@+=feaafiart1ev1aaatCvAUfKttLearuWrP9MDH5MBPbIqV92AaeXatLxBI9gBaebbnrfifHhDYfgasaacH8akY=wiFfYdH8Gipec8Eeeu0xXdbba9frFj0=OqFfea0dXdd9vqai=hGuQ8kuc9pgc9s8qqaq=dirpe0xb9q8qiLsFr0=vr0=vr0dc8meaabaqaciaacaGaaeqabaqabeGadaaakeaaiiGacuWFZoWzgaqeaaaa@2E72@_*h*_(*v*, *v*). It fluctuates as a function of the separation distance *h *since the size of the areas used in semivariogram computation varies as a function of the distance between them: smaller areas tend to be paired at shorter distances, while larger areas can only be paired for distances that exceed half their minimum dimension. This quantity is estimated as the arithmetical average of within-area semivariogram values for any pair of areas separated by a given distance *h*:

γ¯h(v,v)=12N(h)∑α=1N(h)[γ¯(vα,vα)+γ¯(vα+h,vα+h)]     (Equation 21)
 MathType@MTEF@5@5@+=feaafiart1ev1aaatCvAUfKttLearuWrP9MDH5MBPbIqV92AaeXatLxBI9gBaebbnrfifHhDYfgasaacH8akY=wiFfYdH8Gipec8Eeeu0xXdbba9frFj0=OqFfea0dXdd9vqai=hGuQ8kuc9pgc9s8qqaq=dirpe0xb9q8qiLsFr0=vr0=vr0dc8meaabaqaciaacaGaaeqabaqabeGadaaakeaaiiGacuWFZoWzgaqeamaaBaaaleaacqWGObaAaeqaaOGaeiikaGIaemODayNaeiilaWIaemODayNaeiykaKIaeyypa0ZaaSaaaeaacqaIXaqmaeaacqaIYaGmcqWGobGtcqGGOaakcqWGObaAcqGGPaqkaaWaaabCaeaacqGGBbWwcuWFZoWzgaqeaiabcIcaOiabdAha2naaBaaaleaacqWFXoqyaeqaaOGaeiilaWIaemODay3aaSbaaSqaaiab=f7aHbqabaGccqGGPaqkcqGHRaWkcuWFZoWzgaqeaiabcIcaOiabdAha2naaBaaaleaacqWFXoqycqGHRaWkcqWGObaAaeqaaOGaeiilaWIaemODay3aaSbaaSqaaiab=f7aHjabgUcaRiabdIgaObqabaGccqGGPaqkcqGGDbqxaSqaaiab=f7aHjabg2da9iabigdaXaqaaiabd6eaojabcIcaOiabdIgaOjabcMcaPaqdcqGHris5aOGaaCzcaiaaxMaadaqadaqaaiabbweafjabbghaXjabbwha1jabbggaHjabbsha0jabbMgaPjabb+gaVjabb6gaUjabbccaGiabikdaYiabigdaXaGaayjkaiaawMcaaaaa@746F@

where γ¯
 MathType@MTEF@5@5@+=feaafiart1ev1aaatCvAUfKttLearuWrP9MDH5MBPbIqV92AaeXatLxBI9gBaebbnrfifHhDYfgasaacH8akY=wiFfYdH8Gipec8Eeeu0xXdbba9frFj0=OqFfea0dXdd9vqai=hGuQ8kuc9pgc9s8qqaq=dirpe0xb9q8qiLsFr0=vr0=vr0dc8meaabaqaciaacaGaaeqabaqabeGadaaakeaaiiGacuWFZoWzgaqeaaaa@2E72@(*v*_*α*_, *v*_*α*_) and γ¯
 MathType@MTEF@5@5@+=feaafiart1ev1aaatCvAUfKttLearuWrP9MDH5MBPbIqV92AaeXatLxBI9gBaebbnrfifHhDYfgasaacH8akY=wiFfYdH8Gipec8Eeeu0xXdbba9frFj0=OqFfea0dXdd9vqai=hGuQ8kuc9pgc9s8qqaq=dirpe0xb9q8qiLsFr0=vr0=vr0dc8meaabaqaciaacaGaaeqabaqabeGadaaakeaaiiGacuWFZoWzgaqeaaaa@2E72@(*v*_*α*+*h*_, *v*_*α*+*h*_) are computed using Equation (20) with h = 0.

The deconvolution procedure starts with the choice of an initial point-support model *γ*^(0)^(**h**); for example the model *γ*_R_(**h**) fitted to the areal data. This model is regularized according to expression (18), and the theoretically regularized model, γv(0)
 MathType@MTEF@5@5@+=feaafiart1ev1aaatCvAUfKttLearuWrP9MDH5MBPbIqV92AaeXatLxBI9gBaebbnrfifHhDYfgasaacH8akY=wiFfYdH8Gipec8Eeeu0xXdbba9frFj0=OqFfea0dXdd9vqai=hGuQ8kuc9pgc9s8qqaq=dirpe0xb9q8qiLsFr0=vr0=vr0dc8meaabaqaciaacaGaaeqabaqabeGadaaakeaaiiGacqWFZoWzdaqhaaWcbaGaemODayhabaGaeiikaGIaeGimaaJaeiykaKcaaaaa@329C@(*h*), is compared to the model inferred from areal data, *γ*_R_(**h**). The optimization criterion is the relative difference between the two curves, denoted *D*. A new candidate point-support semivariogram *γ*^(1)^(**h**) is derived by rescaling of the initial point-support model *γ*^(0)^(**h**), and then regularized according to expression (18). Model *γ*^(1)^(**h**) becomes the new optimum if it lowers the deviation between the theoretically regularized semivariogram model γv(1)
 MathType@MTEF@5@5@+=feaafiart1ev1aaatCvAUfKttLearuWrP9MDH5MBPbIqV92AaeXatLxBI9gBaebbnrfifHhDYfgasaacH8akY=wiFfYdH8Gipec8Eeeu0xXdbba9frFj0=OqFfea0dXdd9vqai=hGuQ8kuc9pgc9s8qqaq=dirpe0xb9q8qiLsFr0=vr0=vr0dc8meaabaqaciaacaGaaeqabaqabeGadaaakeaaiiGacqWFZoWzdaqhaaWcbaGaemODayhabaGaeiikaGIaeGymaeJaeiykaKcaaaaa@329E@(*h*) and the model fitted to areal data, that is if *D*^(1) ^<*D*^(0)^. Rescaling coefficients are then updated to account for the difference between γv(1)
 MathType@MTEF@5@5@+=feaafiart1ev1aaatCvAUfKttLearuWrP9MDH5MBPbIqV92AaeXatLxBI9gBaebbnrfifHhDYfgasaacH8akY=wiFfYdH8Gipec8Eeeu0xXdbba9frFj0=OqFfea0dXdd9vqai=hGuQ8kuc9pgc9s8qqaq=dirpe0xb9q8qiLsFr0=vr0=vr0dc8meaabaqaciaacaGaaeqabaqabeGadaaakeaaiiGacqWFZoWzdaqhaaWcbaGaemODayhabaGaeiikaGIaeGymaeJaeiykaKcaaaaa@329E@(*h*) and *γ*_R_(**h**), leading to a new candidate model *γ*^(2)^(**h**) for the next iteration. The procedure stops when the maximum number of allowed iterations has been tried (e.g. 35 in this paper) or the decrease in the *D *statistic becomes negligible from one iteration to the next. The use of lag-specific rescaling coefficients provides enough flexibility to modify the initial shape of the point-support semivariogram and makes the deconvolution insensitive to the initial solution adopted.

### Simple approaches to perform area-to-point kriging

A couple of geostatistical approaches have been proposed in the health science literature to derive continuous maps of health outcomes from areal data. The most straightforward method is to interpolate the raw rates to the nodes of a regular grid using point ordinary kriging [2]:

z^OK(us)=∑i=1Kλi(us)z(ui)     (Equation 22)
 MathType@MTEF@5@5@+=feaafiart1ev1aaatCvAUfKttLearuWrP9MDH5MBPbIqV92AaeXatLxBI9gBaebbnrfifHhDYfgasaacH8akY=wiFfYdH8Gipec8Eeeu0xXdbba9frFj0=OqFfea0dXdd9vqai=hGuQ8kuc9pgc9s8qqaq=dirpe0xb9q8qiLsFr0=vr0=vr0dc8meaabaqaciaacaGaaeqabaqabeGadaaakeaacuWG6bGEgaqcamaaBaaaleaacqWGpbWtcqWGlbWsaeqaaOGaeiikaGccbeGae8xDau3aaSbaaSqaaiabdohaZbqabaGccqGGPaqkcqGH9aqpdaaeWbqaaGGaciab+T7aSnaaBaaaleaacqWGPbqAaeqaaaqaaiabdMgaPjabg2da9iabigdaXaqaaiabdUealbqdcqGHris5aOGaeiikaGIae8xDau3aaSbaaSqaaiabdohaZbqabaGccqGGPaqkcqWG6bGEcqGGOaakcqWF1bqDdaWgaaWcbaGaemyAaKgabeaakiabcMcaPiaaxMaacaWLjaWaaeWaaeaacqqGfbqrcqqGXbqCcqqG1bqDcqqGHbqycqqG0baDcqqGPbqAcqqGVbWBcqqGUbGBcqqGGaaicqaIYaGmcqaIYaGmaiaawIcacaGLPaaaaaa@5BAE@

The corresponding kriging variance is computed as:

σOK2(us)=C(0)−∑i=1Kλi(us)C(ui−us)−μ(us)     (Equation 23)
 MathType@MTEF@5@5@+=feaafiart1ev1aaatCvAUfKttLearuWrP9MDH5MBPbIqV92AaeXatLxBI9gBaebbnrfifHhDYfgasaacH8akY=wiFfYdH8Gipec8Eeeu0xXdbba9frFj0=OqFfea0dXdd9vqai=hGuQ8kuc9pgc9s8qqaq=dirpe0xb9q8qiLsFr0=vr0=vr0dc8meaabaqaciaacaGaaeqabaqabeGadaaakeaaiiGacqWFdpWCdaqhaaWcbaGaem4ta8Kaem4saSeabaGaeGOmaidaaOGaeiikaGccbeGae4xDau3aaSbaaSqaaiabdohaZbqabaGccqGGPaqkcqGH9aqpcqWGdbWqcqGGOaakcqaIWaamcqGGPaqkcqGHsisldaaeWbqaaiab=T7aSnaaBaaaleaacqWGPbqAaeqaaaqaaiabdMgaPjabg2da9iabigdaXaqaaiabdUealbqdcqGHris5aOGaeiikaGIae4xDau3aaSbaaSqaaiabdohaZbqabaGccqGGPaqkcqWGdbWqcqGGOaakcqGF1bqDdaWgaaWcbaGaemyAaKgabeaakiabgkHiTiab+vha1naaBaaaleaacqWGZbWCaeqaaOGaeiykaKIaeyOeI0Iae8hVd0MaeiikaGIae4xDau3aaSbaaSqaaiabdohaZbqabaGccqGGPaqkcaWLjaGaaCzcamaabmaabaGaeeyrauKaeeyCaeNaeeyDauNaeeyyaeMaeeiDaqNaeeyAaKMaee4Ba8MaeeOBa4MaeeiiaaIaeGOmaiJaeG4mamdacaGLOaGaayzkaaaaaa@6C63@

The weights *λ*_*i*_(**u**_*s*_) are the solution of the following ordinary kriging system:

∑j=1Kλj(us)C(ui−uj)+μ(us)=C(ui−us)i=1,...,K∑j=1Kλj(us)=1     (Equation 24)
 MathType@MTEF@5@5@+=feaafiart1ev1aaatCvAUfKttLearuWrP9MDH5MBPbIqV92AaeXatLxBI9gBaebbnrfifHhDYfgasaacH8akY=wiFfYdH8Gipec8Eeeu0xXdbba9frFj0=OqFfea0dXdd9vqai=hGuQ8kuc9pgc9s8qqaq=dirpe0xb9q8qiLsFr0=vr0=vr0dc8meaabaqaciaacaGaaeqabaqabeGadaaakeaafaqaaeGacaaabaWaaabCaeaaiiGacqWF7oaBdaWgaaWcbaGaemOAaOgabeaaaeaacqWGQbGAcqGH9aqpcqaIXaqmaeaacqWGlbWsa0GaeyyeIuoakiabcIcaOGqabiab+vha1naaBaaaleaacqWGZbWCaeqaaOGaeiykaKIaem4qamKaeiikaGIae4xDau3aaSbaaSqaaiabdMgaPbqabaGccqGHsislcqGF1bqDdaWgaaWcbaGaemOAaOgabeaakiabcMcaPiabgUcaRiab=X7aTjabcIcaOiab+vha1naaBaaaleaacqWGZbWCaeqaaOGaeiykaKIaeyypa0Jaem4qamKaeiikaGIae4xDau3aaSbaaSqaaiabdMgaPbqabaGccqGHsislcqGF1bqDdaWgaaWcbaGaem4CamhabeaakiabcMcaPaqaaiabbMgaPjabg2da9iabigdaXiabcYcaSiabc6caUiabc6caUiabc6caUiabcYcaSiabdUealbqaamaaqahabaGae83UdW2aaSbaaSqaaiabdQgaQbqabaaabaGaemOAaOMaeyypa0JaeGymaedabaGaem4saSeaniabggHiLdGccqGGOaakcqGF1bqDdaWgaaWcbaGaem4CamhabeaakiabcMcaPiabg2da9iabigdaXaqaaaaacaWLjaGaaCzcamaabmaabaGaeeyrauKaeeyCaeNaeeyDauNaeeyyaeMaeeiDaqNaeeyAaKMaee4Ba8MaeeOBa4MaeeiiaaIaeGOmaiJaeGinaqdacaGLOaGaayzkaaaaaa@810D@

The covariance C(**h**) is derived from the model fitted to the experimental semivariogram of raw rates that is calculated as:

γ^(h)=12N(h)∑α=1N(h)[z(uα)−z(uα+h)]2     (Equation 25)
 MathType@MTEF@5@5@+=feaafiart1ev1aaatCvAUfKttLearuWrP9MDH5MBPbIqV92AaeXatLxBI9gBaebbnrfifHhDYfgasaacH8akY=wiFfYdH8Gipec8Eeeu0xXdbba9frFj0=OqFfea0dXdd9vqai=hGuQ8kuc9pgc9s8qqaq=dirpe0xb9q8qiLsFr0=vr0=vr0dc8meaabaqaciaacaGaaeqabaqabeGadaaakeaaiiGacuWFZoWzgaqcaiabcIcaOGqabiab+HgaOjabcMcaPiabg2da9maalaaabaGaeGymaedabaGaeGOmaiJaemOta4KaeiikaGIae4hAaGMaeiykaKcaamaaqahabaGaei4waSLaemOEaONaeiikaGIae4xDau3aaSbaaSqaaiab=f7aHbqabaGccqGGPaqkcqGHsislcqWG6bGEcqGGOaakcqGF1bqDdaWgaaWcbaGae8xSdegabeaakiabgUcaRiab+HgaOjabcMcaPiabc2faDnaaCaaaleqabaGaeGOmaidaaaqaaiab=f7aHjabg2da9iabigdaXaqaaiabd6eaojabcIcaOiab+HgaOjabcMcaPaqdcqGHris5aOGaaCzcaiaaxMaadaqadaqaaiabbweafjabbghaXjabbwha1jabbggaHjabbsha0jabbMgaPjabb+gaVjabb6gaUjabbccaGiabikdaYiabiwda1aGaayjkaiaawMcaaaaa@6677@

This approach overlooks two critical facts: 1) rates can be very unreliable if recorded over sparsely populated geographical units, and 2) the spatial support of the data (i.e. county) can not be simply equalled to the prediction support (i.e. grid node). In other words, it is unrealistic to collapse vastly different administrative units into their geographic centroids. Another limitation of this approach is its lack of coherence: there is no guarantee that the average of kriging estimates for all *P*_*α *_points discretizing the county *v*_*α *_yields the areal data *z*(*v*_*α*_):

z(vα)≠1Pα∑s=1Pαz^OK(us)     (Equation 26)
 MathType@MTEF@5@5@+=feaafiart1ev1aaatCvAUfKttLearuWrP9MDH5MBPbIqV92AaeXatLxBI9gBaebbnrfifHhDYfgasaacH8akY=wiFfYdH8Gipec8Eeeu0xXdbba9frFj0=OqFfea0dXdd9vqai=hGuQ8kuc9pgc9s8qqaq=dirpe0xb9q8qiLsFr0=vr0=vr0dc8meaabaqaciaacaGaaeqabaqabeGadaaakeaacqWG6bGEcqGGOaakcqWG2bGDdaWgaaWcbaacciGae8xSdegabeaakiabcMcaPiabgcMi5oaalaaabaGaeGymaedabaGaemiuaa1aaSbaaSqaaiab=f7aHbqabaaaaOWaaabCaeaacuWG6bGEgaqcamaaBaaaleaacqWGpbWtcqWGlbWsaeqaaaqaaiabdohaZjabg2da9iabigdaXaqaaiabdcfaqnaaBaaameaacqWFXoqyaeqaaaqdcqGHris5aOGaeiikaGccbeGae4xDau3aaSbaaSqaaiabdohaZbqabaGccqGGPaqkcaWLjaGaaCzcamaabmaabaGaeeyrauKaeeyCaeNaeeyDauNaeeyyaeMaeeiDaqNaeeyAaKMaee4Ba8MaeeOBa4MaeeiiaaIaeGOmaiJaeGOnaydacaGLOaGaayzkaaaaaa@5A94@

To correct for the "small number problem", Berke [3] proposed to replace the raw rates z(**u**_i_) in Equations (22) and (25) by rates r^
 MathType@MTEF@5@5@+=feaafiart1ev1aaatCvAUfKttLearuWrP9MDH5MBPbIqV92AaeXatLxBI9gBaebbnrfifHhDYfgasaacH8akY=wiFfYdH8Gipec8Eeeu0xXdbba9frFj0=OqFfea0dXdd9vqai=hGuQ8kuc9pgc9s8qqaq=dirpe0xb9q8qiLsFr0=vr0=vr0dc8meaabaqaciaacaGaaeqabaqabeGadaaakeaacuWGYbGCgaqcaaaa@2E29@_*GBS*_(**u**_*i*_) obtained using global empirical Bayes smoothers. Each smoothed rate is easily computed as a linear combination of the raw rate z(**u**_i_) and the global mean m*:

r^
 MathType@MTEF@5@5@+=feaafiart1ev1aaatCvAUfKttLearuWrP9MDH5MBPbIqV92AaeXatLxBI9gBaebbnrfifHhDYfgasaacH8akY=wiFfYdH8Gipec8Eeeu0xXdbba9frFj0=OqFfea0dXdd9vqai=hGuQ8kuc9pgc9s8qqaq=dirpe0xb9q8qiLsFr0=vr0=vr0dc8meaabaqaciaacaGaaeqabaqabeGadaaakeaacuWGYbGCgaqcaaaa@2E29@_*GBS*_(**u**_*i*_) = *λ*(**u**_*i*_)*z*(**u**_*i*_) + [1 - *λ*(**u**_*i*_)]m*     *i *= 1,...,*N *    (Equation 27)

The Bayes shrinkage factor *λ*(**u**_i_) is computed as:

λ(ui)={s2−m∗/n¯s2−m∗/n¯+m∗/n(ui)if s2≥m∗/n¯0otherwise     (Equation 28)
 MathType@MTEF@5@5@+=feaafiart1ev1aaatCvAUfKttLearuWrP9MDH5MBPbIqV92AaeXatLxBI9gBaebbnrfifHhDYfgasaacH8akY=wiFfYdH8Gipec8Eeeu0xXdbba9frFj0=OqFfea0dXdd9vqai=hGuQ8kuc9pgc9s8qqaq=dirpe0xb9q8qiLsFr0=vr0=vr0dc8meaabaqaciaacaGaaeqabaqabeGadaaakeaaiiGacqWF7oaBcqGGOaakieqacqGF1bqDdaWgaaWcbaGaemyAaKgabeaakiabcMcaPiabg2da9maaceqabaqbaeqabiGaaaqaamaalaaabaGaem4Cam3aaWbaaSqabeaacqaIYaGmaaGccqGHsislcqWGTbqBdaahaaWcbeqaaiabgEHiQaaakiabc+caViqbd6gaUzaaraaabaGaem4Cam3aaWbaaSqabeaacqaIYaGmaaGccqGHsislcqWGTbqBdaahaaWcbeqaaiabgEHiQaaakiabc+caViqbd6gaUzaaraGaey4kaSIaemyBa02aaWbaaSqabeaacqGHxiIkaaGccqGGVaWlcqWGUbGBcqGGOaakcqGF1bqDdaWgaaWcbaGaemyAaKgabeaakiabcMcaPaaaaeaacqqGPbqAcqqGMbGzcqqGGaaicqGIZbWCdaahaaWcbeqaaiabkkdaYaaakiabgwMiZkabd2gaTnaaCaaaleqabaGaey4fIOcaaOGaei4la8IafmOBa4MbaebaaeaacqaIWaamaeaacqqGVbWBcqqG0baDcqqGObaAcqqGLbqzcqqGYbGCcqqG3bWDcqqGPbqAcqqGZbWCcqqGLbqzaaaacaGL7baacaWLjaGaaCzcamaabmaabaGaeeyrauKaeeyCaeNaeeyDauNaeeyyaeMaeeiDaqNaeeyAaKMaee4Ba8MaeeOBa4MaeeiiaaIaeGOmaiJaeGioaGdacaGLOaGaayzkaaaaaa@7B1B@

where m* and s^2 ^are the population-weighted sample mean and variance of rates, and n¯
 MathType@MTEF@5@5@+=feaafiart1ev1aaatCvAUfKttLearuWrP9MDH5MBPbIqV92AaeXatLxBI9gBaebbnrfifHhDYfgasaacH8akY=wiFfYdH8Gipec8Eeeu0xXdbba9frFj0=OqFfea0dXdd9vqai=hGuQ8kuc9pgc9s8qqaq=dirpe0xb9q8qiLsFr0=vr0=vr0dc8meaabaqaciaacaGaaeqabaqabeGadaaakeaacuWGUbGBgaqeaaaa@2E29@ is the average population size across the study area. Whenever the rate z(**u**_i_) is based on small population sizes n(**u**_i_) relative to the average size n¯
 MathType@MTEF@5@5@+=feaafiart1ev1aaatCvAUfKttLearuWrP9MDH5MBPbIqV92AaeXatLxBI9gBaebbnrfifHhDYfgasaacH8akY=wiFfYdH8Gipec8Eeeu0xXdbba9frFj0=OqFfea0dXdd9vqai=hGuQ8kuc9pgc9s8qqaq=dirpe0xb9q8qiLsFr0=vr0=vr0dc8meaabaqaciaacaGaaeqabaqabeGadaaakeaacuWGUbGBgaqeaaaa@2E29@, the factor *λ*(**u**_i_) is small and the Bayesian estimate (Equation 27) is close to the global mean m*.

The kriging of empirical Bayesian smoothed rates suffers from the same shortcomings as the kriging of raw rates: lack of coherence and ignorance of the spatial support of the data. To attenuate the smoothing effect caused by the use of a global mean in the Bayes smoother (Equation 27), in this paper kriging was also applied to local Bayes estimates. Local Bayes smoothers are calculated similarly except that all the statistics (i.e. the population-weighted sample mean and variance, population size) are computed within local search windows [29]; for example using the *K *neighboring observed rates. The estimator thus becomes:

r^
 MathType@MTEF@5@5@+=feaafiart1ev1aaatCvAUfKttLearuWrP9MDH5MBPbIqV92AaeXatLxBI9gBaebbnrfifHhDYfgasaacH8akY=wiFfYdH8Gipec8Eeeu0xXdbba9frFj0=OqFfea0dXdd9vqai=hGuQ8kuc9pgc9s8qqaq=dirpe0xb9q8qiLsFr0=vr0=vr0dc8meaabaqaciaacaGaaeqabaqabeGadaaakeaacuWGYbGCgaqcaaaa@2E29@_*LBS*_(**u**_*i*_) = *λ*(**u**_*i*_)*z*(**u**_*i*_) + [1 - *λ*(**u**_*i*_)]m* (**u**_*i*_)     (Equation 29)

where m*(**u**_*i*_) is the population-weighted average of the rates within the search window. The Bayes shrinkage factor *λ*(**u**_i_) is now computed as:

λ(ui)={s2(ui)−m∗(ui)/n¯(ui)s2(ui)−m∗(ui)/n¯(ui)+m∗(ui)/n(ui)if s2(ui)≥m∗(ui)/n¯(ui)0otherwise
 MathType@MTEF@5@5@+=feaafiart1ev1aaatCvAUfKttLearuWrP9MDH5MBPbIqV92AaeXatLxBI9gBaebbnrfifHhDYfgasaacH8akY=wiFfYdH8Gipec8Eeeu0xXdbba9frFj0=OqFfea0dXdd9vqai=hGuQ8kuc9pgc9s8qqaq=dirpe0xb9q8qiLsFr0=vr0=vr0dc8meaabaqaciaacaGaaeqabaqabeGadaaakeaaiiGacqWF7oaBcqGGOaakieqacqGF1bqDdaWgaaWcbaGaemyAaKgabeaakiabcMcaPiabg2da9maaceqabaqbaeqabiGaaaqaamaalaaabaGaem4Cam3aaWbaaSqabeaacqaIYaGmaaGccqGGOaakcqGF1bqDdaWgaaWcbaGaemyAaKgabeaakiabcMcaPiabgkHiTiabd2gaTnaaCaaaleqabaGaey4fIOcaaOGaeiikaGIae4xDau3aaSbaaSqaaiabdMgaPbqabaGccqGGPaqkcqGGVaWlcuWGUbGBgaqeaiabcIcaOiab+vha1naaBaaaleaacqWGPbqAaeqaaOGaeiykaKcabaGaem4Cam3aaWbaaSqabeaacqaIYaGmaaGccqGGOaakcqGF1bqDdaWgaaWcbaGaemyAaKgabeaakiabcMcaPiabgkHiTiabd2gaTnaaCaaaleqabaGaey4fIOcaaOGaeiikaGIae4xDau3aaSbaaSqaaiabdMgaPbqabaGccqGGPaqkcqGGVaWlcuWGUbGBgaqeaiabcIcaOiab+vha1naaBaaaleaacqWGPbqAaeqaaOGaeiykaKIaey4kaSIaemyBa02aaWbaaSqabeaacqGHxiIkaaGccqGGOaakcqGF1bqDdaWgaaWcbaGaemyAaKgabeaakiabcMcaPiabc+caViabd6gaUjabcIcaOiab+vha1naaBaaaleaacqWGPbqAaeqaaOGaeiykaKcaaaqaaiabbMgaPjabbAgaMjabbccaGiabdohaZnaaCaaaleqabaGaeGOmaidaaOGaeiikaGIae4xDau3aaSbaaSqaaiabdMgaPbqabaGccqGGPaqkcqGHLjYScqWGTbqBdaahaaWcbeqaaiabgEHiQaaakiabcIcaOiab+vha1naaBaaaleaacqWGPbqAaeqaaOGaeiykaKIaei4la8IafmOBa4MbaebacqGGOaakcqGF1bqDdaWgaaWcbaGaemyAaKgabeaakiabcMcaPaqaaiabicdaWaqaaiabb+gaVjabbsha0jabbIgaOjabbwgaLjabbkhaYjabbEha3jabbMgaPjabbohaZjabbwgaLbaaaiaawUhaaaaa@99B4@

As for global Bayes smoothers, the relative weight *λ*(**u**_i_) assigned to the observed rate z(**u**_i_) is smaller for less densely populated counties. For counties with similar population sizes, the factor *λ*(**u**_i_) is also smaller in regions of greater homogeneity, as measured by a lower local variance s^2^(**u**_i_).

## Results and discussion

### Analysis of lung and cervix cancer data

#### County-level estimates of mortality risk

**Figure 3:**
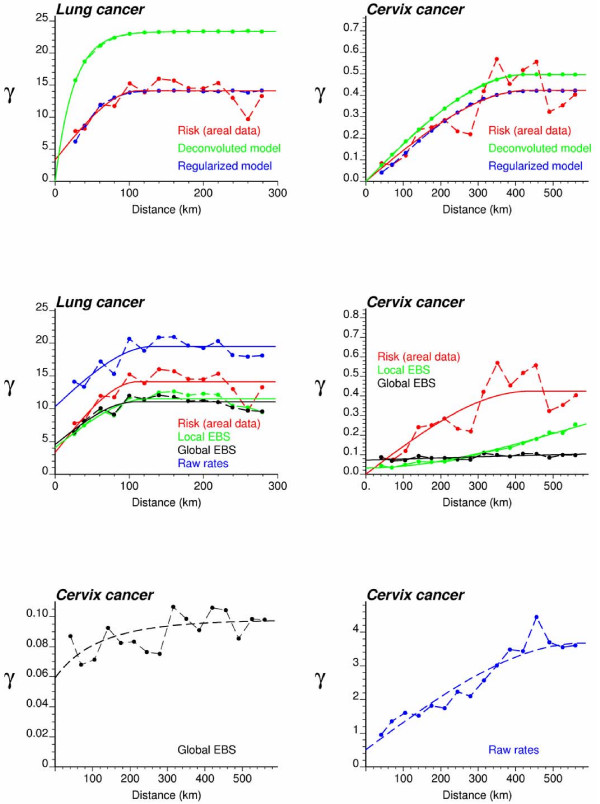
**Semivariogram models used by the different geostatistical approaches for mapping lung and cervix cancer mortality**. The top graphs show the experimental semivariogram of the risk estimated from county-level rate data (red curve), and the results of its deconvolution (green curve). The regularization of the point-support model yields a curve (blue line) that is very close to the experimental one. Bottom graphs show the semivariogram of raw rates, as well as the semivariograms of global and local empirical Bayes estimates.

Figure 3 (top graphs, red curve) shows the omnidirectional semivariograms of lung and cervix cancer mortality risk computed from county-level rates using estimator (5) and the distance measure (1). The experimental semivariogram was fitted using a spherical model with a range of 113 km for lung cancer in Indiana and 437 km for cervix cancer in Region 2. Each model was deconvoluted using the iterative procedure and the high-resolution population maps displayed in Figure 2. For both regions, the procedure stopped once a small (i.e. <1%) decrease in the *D *statistic occurred three times, after 28 iterations for Region 1 and 10 iterations for Region 2. As expected, the point-support semivariogram model (green curve) has a higher sill since the punctual process has a larger variance than its aggregated form. The difference between the sills is particularly large for Region 1, which confirms the stronger impact of regularization for processes with smaller range of autocorrelation [[30], p. 465]. Applying expression (18) to the point-support model yields a theoretically regularized semivariogram model (blue curve) that is close to the one fitted to experimental values, which validates the consistency of the deconvolution.

**Figure 4:**
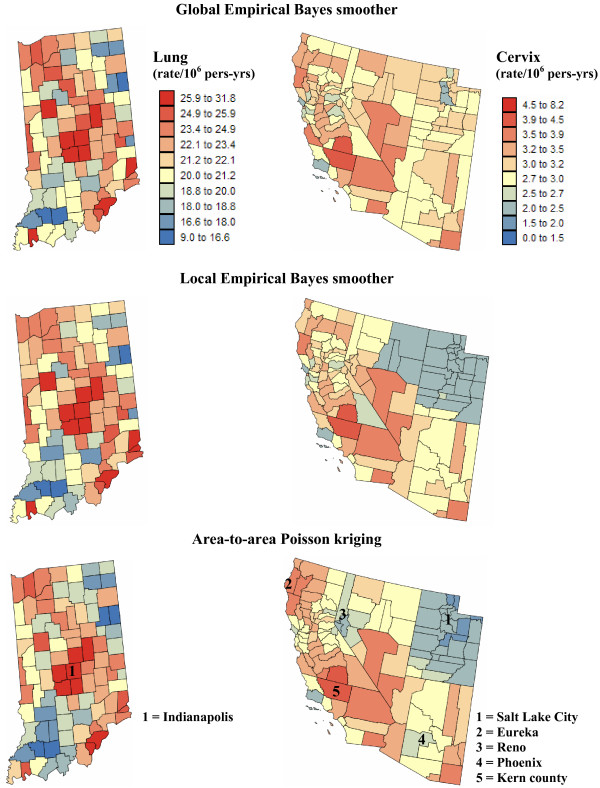
**County maps of lung and cervix cancer mortality risks computed using alternative estimators**. The fill color represents the risk estimated using the following approaches: global and local empirical Bayes smoothers (EBS), and area-to-area (ATA) Poisson kriging. The color legend applies to all the maps of the same region; the class boundaries correspond to the deciles of the histogram of original rates.

The deconvoluted semivariogram models were used to estimate aggregated risk values at the county level in both regions (ATA kriging); see Figure 4 (bottom maps). Figure 4 also shows the maps produced by global and local empirical Bayes smoothers. In all cases, the estimation was based on *K *= 32 closest observations which were selected according to the population-weighted distance between counties for ATA kriging and the Euclidian distance between the county population-weighted centroids for the two other methods. All maps are smoother than the map of raw rates since the noise due to small population sizes is filtered. The smoothing is particularly strong for the less frequent cervix cancer with a one order of magnitude drop in the variance; see Table 1 (top rows). The variance of smoothed mortality rates is half the variance of raw rates for lung cancer. Table 1 indicates that for both cancers the smoothing is slightly smaller for ATA kriging versus local Bayes smoothers. The global EBS estimates are the least variable, in particular for cervix cancer.

**Table 1:** Summary statistics for county-level and point-level estimates of lung and cervix cancer mortality.

Estimators	Lung cancer			Cervix cancer		
County-level estimates	Mean	Variance	Min-max	Mean	Variance	Min-Max

Observed rates	21.19	18.48	9.071–31.79	2.851	2.446	0.000–8.138
Global EBS	21.95	10.57	13.91–31.68	3.052	0.092	2.281–4.051
Local EBS	22.10	10.79	13.36–31.71	2.838	0.224	2.041–4.014
ATA Poisson kriging	21.58	10.96	13.22–31.56	2.851	0.264	1.921–4.081

Point estimates						

Kriged rates	21.15	4.970	15.46–26.85	2.972	1.003	0.470–6.702
Kriged global EBS	21.90	3.703	17.09–27.92	3.085	0.010	2.731–3.444
Kriged local EBS	22.02	4.520	16.25–28.24	2.883	0.154	2.061–3.519
ATP Poisson kriging	21.22	9.510	12.13–33.90	2.985	0.266	1.906–4.308

Aggregates of point estimates						

Kriged rates	21.22	4.832	16.14–26.29	2.849	0.641	0.955–5.084
Kriged global EBS	21.99	3.529	17.74–27.11	3.052	0.011	2.765–3.314
Kriged local EBS	22.12	4.390	16.94–27.43	2.842	0.157	2.064–3.406
ATP Poisson kriging	21.58	10.96	13.22–31.56	2.851	0.264	1.921–4.081

Mean, variance and range of cancer mortality rates per 100,000 person-years estimated at the county level or at the nodes of a 5 km grid using four alternative methods: point kriging of observed rates, point kriging of global and local empirical Bayesian smoothed (EBS) rates, area-to-area (ATA) and area-to-point (ATP) Poisson kriging. Statistics for the county-level aggregates of point estimates are listed at the bottom of the table.

The three risk maps of Region 1 are relatively similar; see Figure 4 (left column). All methods smoothed low lung cancer rates recorded in a few North-western and North-eastern counties characterized by smaller population sizes. For example, after application of Poisson kriging the minimum rate increased from 9.071 to 13.22 deaths/100,000 habitants; see Table 1. Since the highest lung cancer mortality rates are observed in the most populated counties in Indiana, the maximum rates remain practically the same after smoothing.

Differences between methods are much more pronounced for cervix cancer mortality in Region 2; see Figure 4 (right column). Unlike the case of lung cancer, both high and low cancer rates are smoothed. Except on the local EBS map, the high risk area formed by two central counties in Figure 1 faded, which illustrates the potential pitfalls associated with the interpretation of the map of observed rates. The highest risk (4.081 deaths/100,000 habitants for ATA kriging) is predicted for Kern County, just west of Santa Barbara County (California). Zero cervix cancer mortality rates recorded in sparsely populated counties in Utah were also smoothed, leading to minimum values of 1.92–2.28 deaths/100,000 habitants. Global empirical Bayes method causes a strong smoothing of the raw rates, in particular in Utah where outside Salt Lake City most county rates shrunk towards the global mean of 2.85 per 100,000 person-years. The two other methods (ATA kriging and local EBS) create risk maps that show a large contrast between a low risk cluster in Utah and a high risk cluster in Southern California and Southern Nevada. Still, these two maps exhibit important differences: rates recorded in the vicinity of urban areas (i.e. low rates around Reno and Phoenix, or high rates around Eureka) are smoothed less by ATA kriging. Conversely, more smoothing occurred in sparsely populated Eastern Nevada, leading to higher risk on the ATA kriged map versus local EBS. In summary, ATA kriging seems to weigh more heavily the population size in the filtering of the noisy rate maps.

#### Point-level estimates of mortality risk

To eliminate the visual bias associated with the interpretation of the choropleth maps of Figure 4, continuous risk surfaces were created for lung and cervix cancer mortality. The most straightforward mapping techniques amount at conducting a point kriging of the county-level raw or smoothed rates, which are simply assigned to the population-weighted centroids of those counties. In other words, one implicitly assumes that all the habitants of a county live at the same location and the measured rate thus refers to this specific location. Note that to ensure that both ATP and point kriging incorporate information on the spatial distribution of county population, the population-weighted centroids displayed in Figure 2 (middle maps) were used for semivariogram estimation and point kriging throughout the analysis. These centroids will be referred to as county centroids, as opposed to geographic centroids, hereafter. A prerequisite for kriging is a semivariogram model that is fitted to experimental values computed from centroid-based rate data using estimator (25). Figure 3 (bottom graphs) shows that the largest semivariogram values are observed for raw rates (blue curve), which is expected because of the additional random variability caused by the small number problem. Since cervix cancer is less frequent than lung cancer, its mortality rates are more likely to be impacted by the small number problem and display higher levels of noise, leading to a sill that is one order of magnitude larger than for the other curves (Figure 3, right bottom graph). The semivariogram of empirical Bayes estimates has the smallest values since the smoothing reduces the variance of raw rates, in particular for cervix cancer. The use of local versus global empirical smoothers has little impact on the semivariogram for the frequent lung cancer while, as expected, the global smoothers lead to a much lower sill for the semivariogram of cervix cancer. For that cancer, the semivariogram of local EBS risk estimates (green curve) has a concave shape which reflects the presence of a trend that is apparent on the smoothed risk map of Figure 4 (i.e. low rate in Utah and high rates in Southern California). This semivariogram was modelled using a cubic model [6,31] with a range of 2,041 km, while the semivariogram model for local EBS estimates consists of two exponential models and a large nugget effect; see Figure 3 (left bottom graph).

**Figure 5:**
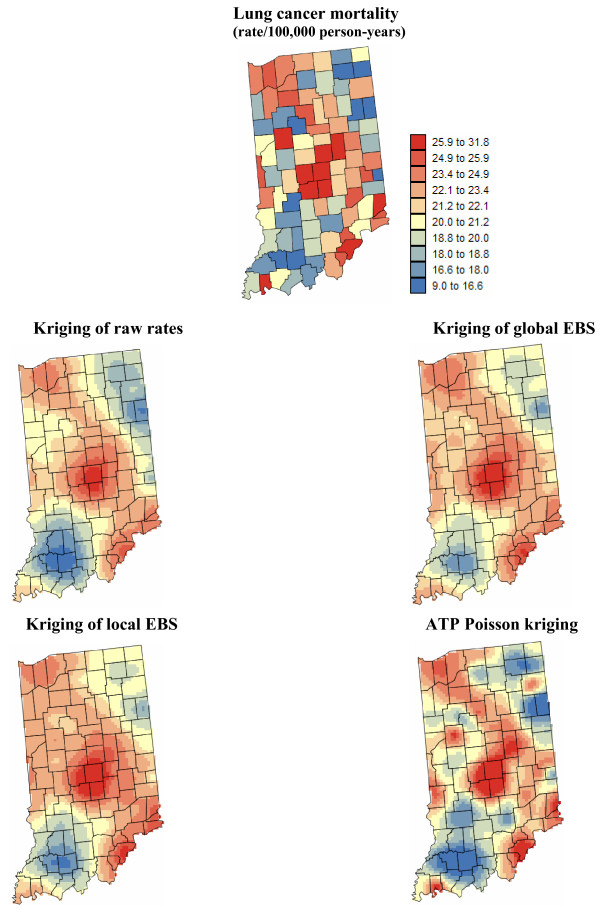
**Map of lung cancer mortality rates in Region 1, and the risk estimated by the different forms of kriging**. The fill color represents the age-adjusted mortality rate per 100,000 person-years recorded over the period 1970–1994 (top graph) or the risk estimated using the following approaches: point kriging of raw rates, point kriging of global and local empirical Bayes smoothers (EBS), and area-to-point (ATP) Poisson kriging. The color legend applies to all the maps; the class boundaries correspond to the deciles of the histogram of original rates.

**Figure 6:**
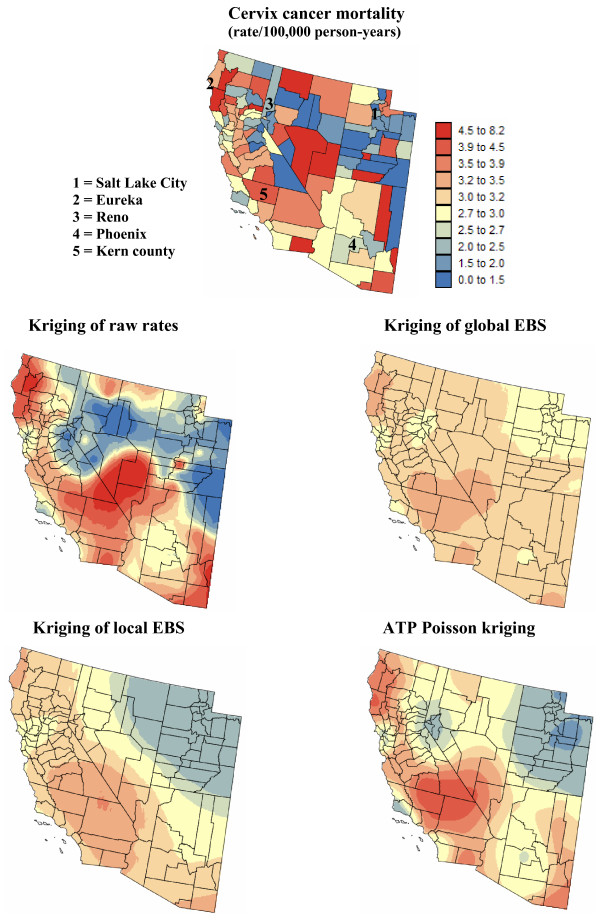
**Map of cervix cancer mortality rates in Region 2, and the risk estimated by the different forms of kriging**. The fill color represents the age-adjusted mortality rate per 100,000 person-years recorded over the period 1970–1994 (top graph) or the risk estimated using the following approaches: point kriging of raw rates, point kriging of global and local empirical Bayes smoothers (EBS), and area-to-point (ATP) Poisson kriging. The color legend applies to all the maps; the class boundaries correspond to the deciles of the histogram of original rates.

Figures 5 and 6 show the risk values estimated at the nodes of a 5 km grid using the following methods: point kriging of raw rates, point kriging of global and local empirical Bayesian smoothed rates (EBS), and area-to-point Poisson kriging (ATP). In all four cases, the estimation was based on *K *= 32 closest county-level rates (local search window). To enforce the coherence constraint, within a given county each grid node was estimated using the same 32 county-level rates; that is the proximity is measured in terms of distance between the centroids of both the prediction and the data counties instead of the distance between the grid node in the prediction county and the centroid of the data county. For lung cancer, the maps created by point kriging of raw and EBS rates show only minor differences; higher EBS risk estimates are found close to the Northeast and Northwest borders where low rates in sparsely populated counties were smoothed by the empirical Bayes method. The ATP kriged map displays much more details, with the presence of clearly delineated areas of lower and higher rates. These visual impressions are confirmed by the statistics listed in Table 1: the variance of the set of ATP kriging estimates (9.510) is twice the variance calculated for the other methods (3.703–4.970). Similar conclusions can be drawn from the comparison of the ATP and EBS kriging maps for Region 2: the variance of ATP kriging estimates (0.266) is twice the variance obtained for the kriging of local EBS (0.154) and more than one order of magnitude larger than for the global empirical Bayes smoother (0.010). The isopleth map of GBS risk estimates is very smooth because of the large nugget effect of the corresponding semivariogram model (recall Figure 3, left bottom graph). Conversely, the kriged map of raw rates is the most variable since it is based on the interpolation of unstable cervix cancer mortality rates, and one can distinguish very clearly the location of several county centroids.

Although the interpretation of spatial patterns is beyond the scope of this methodology paper, comparison of ATP and local EBS kriging maps for Region 2 offers interesting insights about the spatial distribution of cervix cancer mortality risk:

• The impact of low or high rates recorded in the vicinity of urban areas (i.e. low rates around Reno and Phoenix, or high rates around Eureka) is much more apparent on the ATP kriged map. This short-scale variability in the risk map results from the greater weight assigned to population density and areal data through the coherence constraint in area-to-point kriging.

• Lower cervix cancer mortality is clearly confined to Utah on the ATP kriging map, while these low rates expand to Eastern Nevada on the maps of kriged local EBS.

• ATP kriging highlights a cluster of high mortality risk in Southern California. A similar cluster was identified with Kern County on the choropleth map of ATA kriged risk in Figure 4. The isopleth risk map shows that high risks are not confined to this sole county but potentially spread over four counties. This information, which is important for designing prevention strategies, is blurred by the use of county-level estimates.

**Figure 7:**
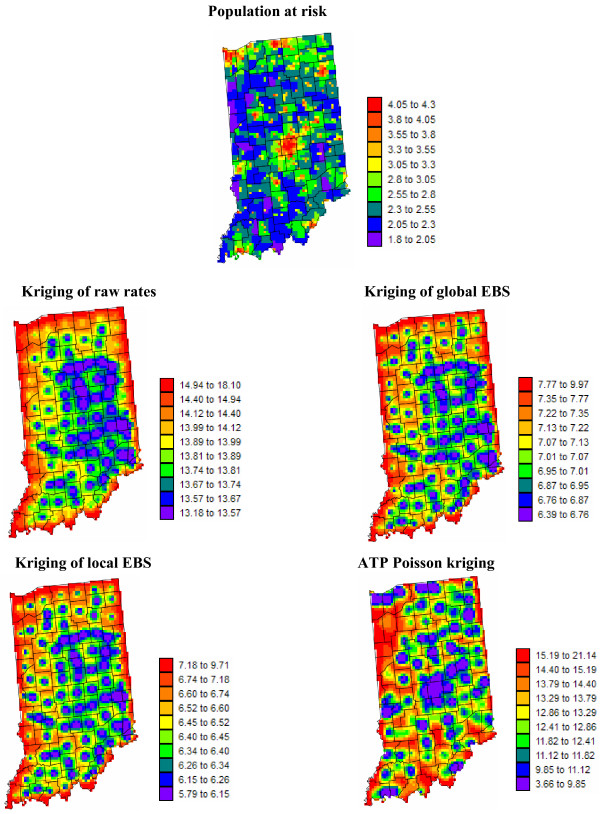
**White female population map for Region 1, and the prediction variance for the different forms of kriging**. The fill color represents the population at risk (lognormal scale) or the kriging variance associated with the risk maps of Figure 5. The following estimation techniques were used: point kriging of raw rates, point kriging of global and local empirical Bayes smoothers (EBS), and area-to-point (ATP) Poisson kriging. The units for the kriging variance maps are (age-adjusted mortality rate per 100,000 person-years)^2^, and the class boundaries correspond to the deciles of the histogram of variance.

**Figure 8:**
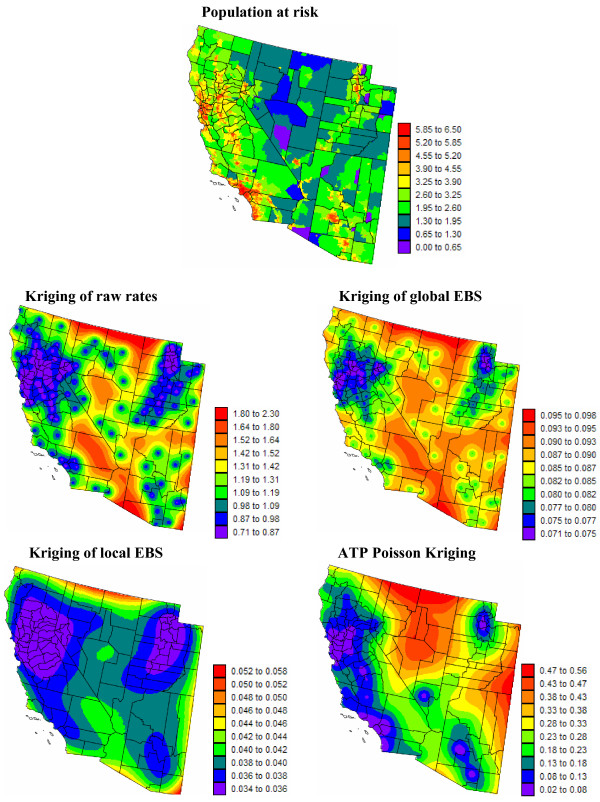
**White female population map for Region 2, and the prediction variance for the different forms of kriging**. The fill color represents the population at risk (lognormal scale) or the kriging variance associated with the risk maps of Figure 6. The following estimation techniques were used: point kriging of raw rates, point kriging of global and local empirical Bayes smoothers (EBS), and area-to-point (ATP) Poisson kriging. The units for the kriging variance maps are (age-adjusted mortality rate per 100,000 person-years)^2^, and the class boundaries correspond to the deciles of the histogram of variance.

Figures 7 and 8 show the maps of the kriging variance associated with the risk estimates of Figures 5 and 6. Differences among interpolation methods are much more pronounced in terms of prediction variance than estimated risk. The point kriging variance is mainly influenced by the location of county centroids (i.e. data geometry): prediction variances are small in the vicinity of county centroids and increase in extrapolation situation, e.g. close to the state borders for the maps of Region 1 (Figure 7). The location of county centroids is particularly obvious on the global EBS map in Region 2 because of the large nugget effect of the corresponding semivariogram. Furthermore, the variance map for local EBS is very smooth as a result of the highly continuous behaviour of the cubic semivariogram model at the origin; recall Figure 3 (right middle graph, green curve). The pattern of these point kriging variance maps essentially reflects the presence of two clusters of small counties (i.e. nearby county centroids) in Northern California and in Utah. The point kriging variance indirectly accounts for the spatial distribution of population within each county through the use of population-weighted centroids, yet differences in population sizes among counties is ignored. This information is incorporated directly into the computation of the ATP kriging variance leading to increased uncertainty in sparsely populated areas. This effect is particularly apparent in Region 2; compare the bottom right map in Figure 8 with the top population map. One can easily associate the location of pockets of low variance with urban centers, such as Los Angeles, San Francisco, Salt Lake City, Las Vegas or Tucson. In both regions, the variance of EBS point risk estimates is the smallest since the smoothing leads to semivariograms with the lowest sills; recall Figure 3.

#### Coherence of estimation techniques

The isopleth risk maps displayed in Figures 5 and 6 can be viewed as the product of the disaggregation of the choropleth maps of Figure 4. One should thus expect that the aggregation of point kriging risk estimates within each county returns the areal data for that county. This "coherence constraint" is not implicit to the point kriging approach, and Table 1 (bottom rows) confirms that the distributions of aggregated point risk estimates and of the county-level rates used in point kriging do not share the same statistics. Because of the smoothing effect of kriging, the variance of aggregated estimates is 50 to 80% smaller than the variance of areal data. This is not the case for ATP kriging where the coherence constraint (Equation 15) ensures that the population-weighted variance, as well as the population-weighted mean, of ATP kriging estimates equals the variance and mean of ATA kriging estimates; see Table 1. This coherence constraint explains the greater level of details noticed in the ATP kriging map versus the risk maps created by the simple point kriging techniques.

#### Point Poisson kriging versus ATA Poisson kriging

**Figure 9:**
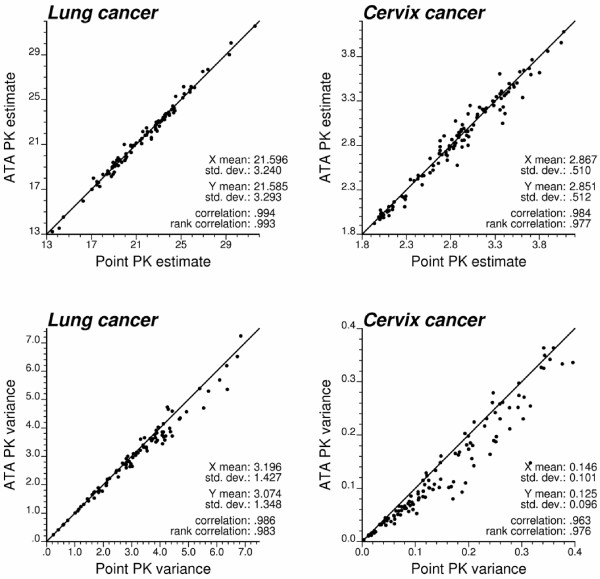
**Impact of ignoring the spatial support and population distribution on Poisson kriging results**. Top scatterplots illustrate the similarity of the cancer mortality risk estimated using punctual and area-to-area (ATA) Poisson kriging. Ignoring the shape and size of counties (i.e. collapsing county rates to their population-weighted centroids) leads to an overestimation of the kriging variance, in particular for the set of vastly different counties in Region 2.

In earlier papers on the use of Poisson kriging for filtering noise in cancer mortality maps [15], a point kriging approach was implemented whereby only county geographic centroids were considered for semivariogram estimation and kriging. To investigate the impact of these simplifying assumptions on the computation of the kriging estimate and variance the results of ATA and point Poisson kriging are compared in Figure 9. Estimation of the mortality risk is fairly robust with respect to the use of centroid geography, in particular for Region 1 where all counties have similar size and shape. Discrepancies between both approaches are much more pronounced for the kriging variance: ignoring the spatial support of county-level data leads almost systematically to larger kriging variance; see Figure 9 (bottom graphs). This result is consistent with the "change-of-support" theory that predicts smaller error variances for block versus point estimation. The magnitude of the overestimation by point kriging is the largest for Region 2 that includes very large counties and has a wide range of county sizes and shapes.

### Simulation studies

Figures 3 through 8 illustrated the major differences between alternative approaches for creating isopleth risk maps from aggregated rates. An objective assessment of the prediction performances of the various techniques requires, however, the availability of the underlying risk maps, which are unknown in practice. Simulation provides a way to generate multiple realizations of the spatial distribution of cancer mortality rates that can be analyzed using alternative approaches. Predicted risks can then be compared to the risk maps used in the simulation.

**Figure 10:**
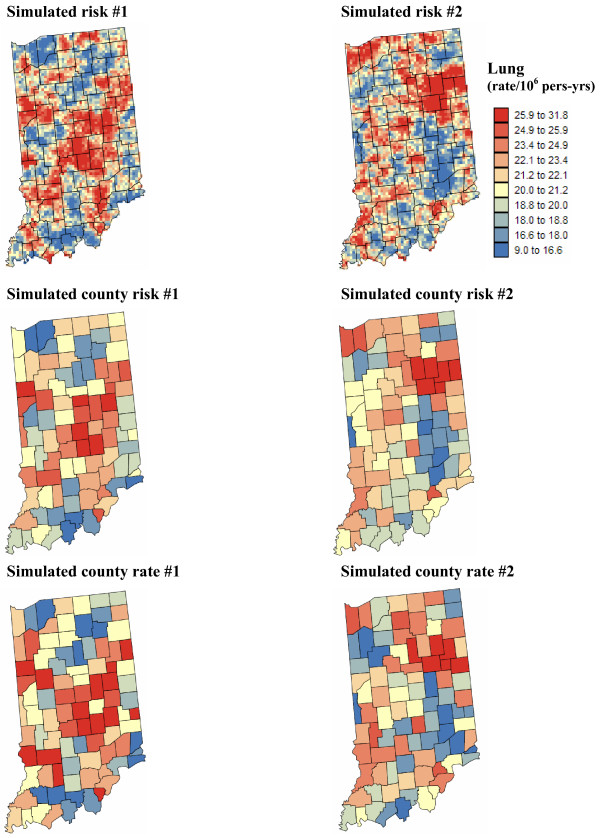
**County maps of lung cancer mortality rates simulated under two scenarios for the underlying continuous risk map**. The number of cases for each county was simulated by random sampling of a Poisson distribution that is defined by the white female population map of Figure 1 and the county-level aggregation of a continuous risk map generated using sequential Gaussian simulation. The units are age-adjusted mortality rates per 100,000 person-years. The color legend applies to all the maps; the class boundaries correspond to the deciles of the histogram of original rates.

**Figure 11:**
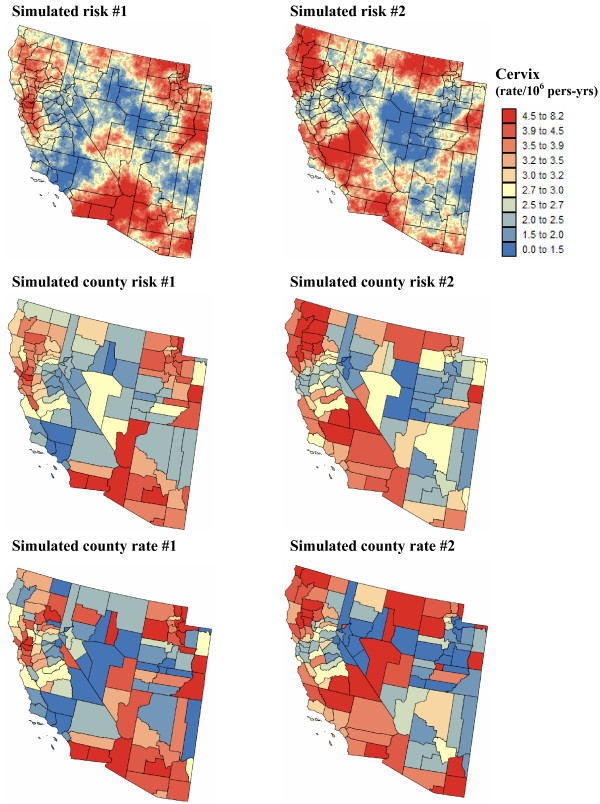
**County maps of cervix cancer mortality rates simulated under two scenarios for the underlying continuous risk map**. The number of cases for each county was simulated by random sampling of a Poisson distribution that is defined by the white female population map of Figure 1 and the county-level aggregation of a continuous risk map generated using sequential Gaussian simulation. The units are age-adjusted mortality rates per 100,000 person-years. The color legend applies to all the maps; the class boundaries correspond to the deciles of the histogram of original rates.

For both lung and cervix cancers, *L *= 100 maps of county-level mortality rates {z^(l)^(*v*_*α*_), *α *= 1,..., N; l = 1,..., L} were simulated using the following procedure:

1. Continuous maps of mortality risk values, {r^(l)^(***u***_s_), s = 1,..., S}, are first simulated using non-conditional sequential Gaussian simulation (see [16,9], p. 380 for a description of the algorithm). The simulation grid, which has a 5 km spacing, consists of S = 3,751 and S = 48,474 nodes for Regions 1 and 2, respectively. Five different risk maps (i.e. L = 5) were generated for each cancer, and two realizations for each region are displayed at the top of Figures 10 and 11. Each realization reproduces the histogram of raw cancer mortality rates, while the deconvoluted semivariogram models of Figure 3 were used in the simulation algorithm: exponential model with a range of 75 km for Region 1 and a spherical model with range of 425 km for Region 2. Both models have a zero nugget effect and a unit sill.

2. Each simulated risk map was combined with the population maps of Figure 2 to compute a number of deaths for all grid nodes. Both simulated death counts and population data were then aggregated within each county, and their ratio defines the simulated county-level mortality risk:

r(l)(vα)=1n(vα)∑s=1Pαn(us)r(l)(us)withn(vα)=∑s=1Pαn(us)     (Equation 30)
 MathType@MTEF@5@5@+=feaafiart1ev1aaatCvAUfKttLearuWrP9MDH5MBPbIqV92AaeXatLxBI9gBaebbnrfifHhDYfgasaacH8akY=wiFfYdH8Gipec8Eeeu0xXdbba9frFj0=OqFfea0dXdd9vqai=hGuQ8kuc9pgc9s8qqaq=dirpe0xb9q8qiLsFr0=vr0=vr0dc8meaabaqaciaacaGaaeqabaqabeGadaaakeaafaqabeqadaaabaGaemOCai3aaWbaaSqabeaacqGGOaakcqWGSbaBcqGGPaqkaaGccqGGOaakcqWG2bGDdaWgaaWcbaacciGae8xSdegabeaakiabcMcaPiabg2da9maalaaabaGaeGymaedabaGaemOBa4MaeiikaGIaemODay3aaSbaaSqaaiab=f7aHbqabaGccqGGPaqkaaWaaabCaeaacqWGUbGBcqGGOaakieqacqGF1bqDdaWgaaWcbaGaem4CamhabeaakiabcMcaPiabdkhaYnaaCaaaleqabaGaeiikaGIaemiBaWMaeiykaKcaaaqaaiabdohaZjabg2da9iabigdaXaqaaiabdcfaqnaaBaaameaacqWFXoqyaeqaaaqdcqGHris5aOGaeiikaGIae4xDau3aaSbaaSqaaiabdohaZbqabaGccqGGPaqkaeaacqqG3bWDcqqGPbqAcqqG0baDcqqGObaAaeaacqWGUbGBcqGGOaakcqWG2bGDdaWgaaWcbaGae8xSdegabeaakiabcMcaPiabg2da9maaqahabaGaemOBa4MaeiikaGIae4xDau3aaSbaaSqaaiabdohaZbqabaGccqGGPaqkaSqaaiabdohaZjabg2da9iabigdaXaqaaiabdcfaqnaaBaaameaacqWFXoqyaeqaaaqdcqGHris5aaaakiaaxMaacaWLjaWaaeWaaeaacqqGfbqrcqqGXbqCcqqG1bqDcqqGHbqycqqG0baDcqqGPbqAcqqGVbWBcqqGUbGBcqqGGaaicqaIZaWmcqaIWaamaiaawIcacaGLPaaaaaa@830E@

The maps of aggregated risk values, {r^(l)^(*v*_*α*_), *α *= 1,..., N}, are displayed in the middle of Figures 10 and 11.

3. For each cancer and each of the five risk maps, 20 realizations of the number of deaths recorded over each county *v*_*α *_were generated by random drawing of a Poisson distribution whose mean parameter is r^(l)^(*v*_*α*_) × n(*v*_*α*_). The division of the simulated death counts by the county population leads to 5 × 20 = 100 sets of simulated mortality rates for each cancer; see bottom of Figures 10 and 11 for the first realization generated for the risk maps displayed in the same figures. The noise caused by the small number problem is particularly apparent for cervix cancer. For example, a couple of counties in the North central part of realization #1 display high mortality rates, while the underlying risk value is low.

### Comparison of prediction performances

Each map of simulated rates {z^(l)^(*v*_*α*_), *α *= 1,..., N} underwent a (geo)statistical analysis in order to estimate point risk values using four alternate approaches: point kriging of raw rates, point kriging of global and local empirical Bayesian smoothed rates (EBS), and area-to-point Poisson kriging (ATP). In all four cases, the estimation at each grid node was based on *K *= 32 closest areal data (local search window). For each realization, the semivariogram needed for each type of kriging was estimated before being automatically modelled and deconvoluted in the case of ATP kriging.

**Figure 12:**
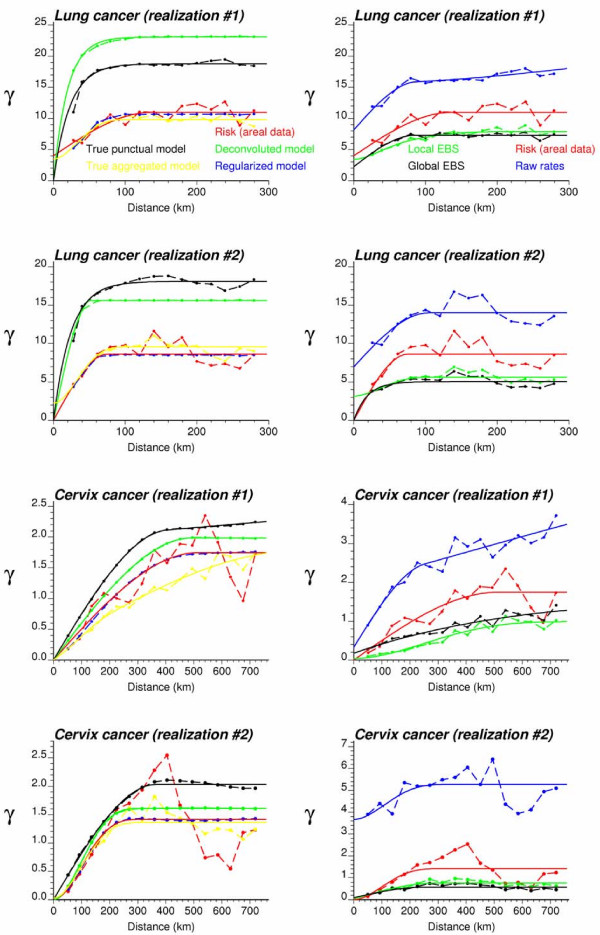
**Semivariogram models inferred from the simulated rate maps of Figures 10 and 11**. The left column shows the experimental semivariogram of the risk estimated from county-level rate data (red curve), and the results of its deconvolution (green curve) which is reasonably close to the "true" point-support model (black curve). The regularization of the point-support model yields a curve (blue line) that is very close to the experimental one. Yellow curves represent the semivariograms computed from the underlying county risk maps. Right column shows the semivariogram of raw rates, as well as the semivariograms of global and local empirical Bayesian smoothed (EBS) rates.

Figure 12 shows the different semivariogram models for the two simulated maps of lung and cervix cancer mortality displayed at the bottom of Figures 10 and 11. The reference point (black curve) and areal (yellow curve) support models are the semivariograms fitted directly to the original simulated risk maps before and after aggregation to the county level; see Figure 12 (left column). Models resulting from the deconvolution procedure are plotted as green curves. Comparison of the green and black solid curves indicates that the deconvolution yields point-support models that are reasonably close to the underlying ones in terms of range values. Each deconvoluted model was regularized according to Equation (18), and the resulting model (blue curve) agrees fairly well with the risk semivariogram model fitted to areal data (red curve), which demonstrates the convergence of the iterative deconvolution procedure. Note also how this risk semivariogram model is close to the reference areal support model (yellow curve). Because of the good agreement between theoretically regularized and the risk semivariogram models inferred from the county-level rates, discrepancies at the point-support level are essentially caused by the use of the regularization formula (Equation 18). An important factor that influences the deconvolution results is the behavior at the origin of the regularized and point-support semivariogram models; for example the fitting of a nugget effect or the use of a spherical (linear behavior) versus cubic (parabolic behavior) model. Unfortunately, in absence of point data this part of the semivariogram model can not be validated. In this paper, no nugget effect was systematically fitted to the point-support model to account for the characteristics of the model used in the simulation.

The semivariogram models used by the point kriging approaches are plotted in the right column of Figure 12. In particular for cervix cancer, the noise caused by the small number problem leads to semivariograms for raw rates that have a high sill and a large relative nugget effect (blue curves). Rate smoothing using global or local empirical Bayes approaches substantially reduces the sample variability, resulting in smaller sills and nuggets effects, as well as parabolic behaviour for most of the semivariograms at the origin (green and black curves). The risk semivariogram estimator (Equation 5), which accounts for population size in the estimation, yields models with intermediate sills (red curves).

**Figure 13:**
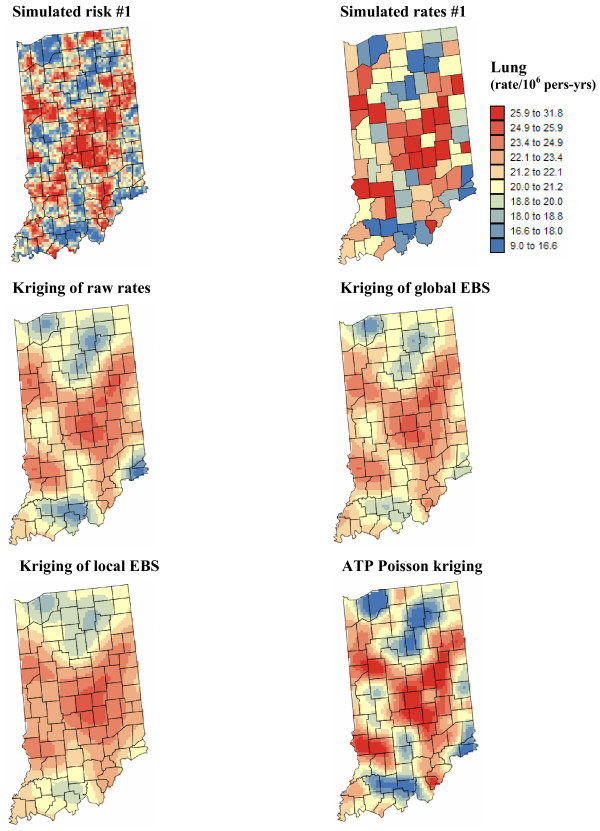
**Simulated lung cancer risk map and the results of the different forms of area-to-point kriging**. The fill color represents mortality risk per 100,000 person-years simulated by sequential Gaussian simulation and the results of the estimation using the following approaches: point kriging of raw rates, point kriging of global and local empirical Bayesian smoothed (EBS) rates, and area-to-point (ATP) Poisson kriging. The color legend applies to all the maps; the class boundaries correspond to the deciles of the histogram of original rates.

**Figure 14:**
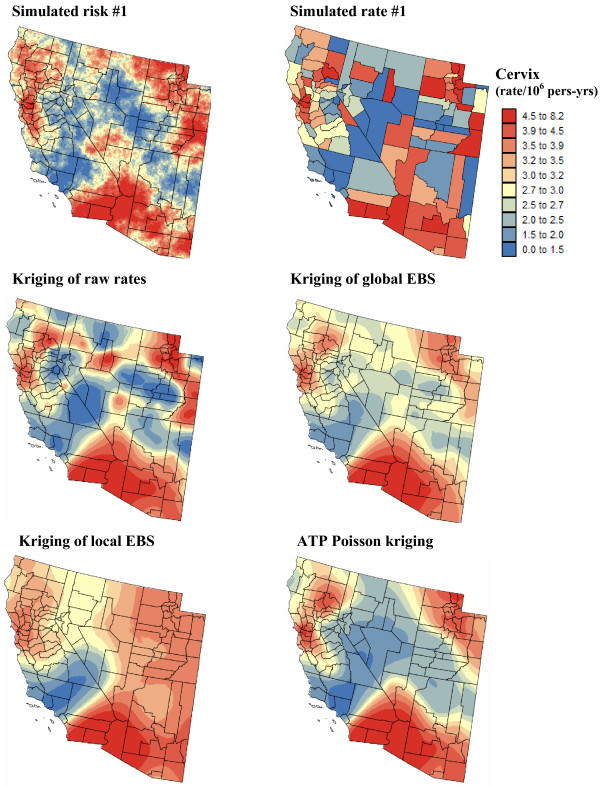
**Simulated cervix cancer risk map and the results of the different forms of area-to-point kriging**. The fill color represents mortality risk per 100,000 person-years simulated by sequential Gaussian simulation and the results of the estimation using the following approaches: point kriging of raw rates, point kriging of global and local empirical Bayesian smoothed (EBS) rates, and area-to-point (ATP) Poisson kriging. The color legend applies to all the maps; the class boundaries correspond to the deciles of the histogram of original rates.

Figures 13 and 14 show, for the simulated rate maps #1 of Figures 10 and 11, the risk values estimated at the nodes of a 5 km grid using the four alternate methods. The analysis of simulated maps confirms the conclusions drawn from the analysis of real cancer mortality rates in Figures 5 and 6. The location of county centroids is the most apparent on the kriged map of raw rates which is also the most variable because it is based on the interpolation of unstable mortality rates, in particular for cervix cancer. Kriging of global and local EBS estimates leads to very smooth maps free of the influence of unreliable rates. Poisson kriging, through its coherence constraint, generates maps of risk estimates that are locally more detailed (i.e. less smoothing) and reveal features of the true risk maps that were blurred by empirical Bayes smoothing, such as the low risk area extending up to the eastern border of Region 2.

For all 100 realizations of each type of cancer, predicted risks {rP(l)
 MathType@MTEF@5@5@+=feaafiart1ev1aaatCvAUfKttLearuWrP9MDH5MBPbIqV92AaeXatLxBI9gBaebbnrfifHhDYfgasaacH8akY=wiFfYdH8Gipec8Eeeu0xXdbba9frFj0=OqFfea0dXdd9vqai=hGuQ8kuc9pgc9s8qqaq=dirpe0xb9q8qiLsFr0=vr0=vr0dc8meaabaqaciaacaGaaeqabaqabeGadaaakeaacqWGYbGCdaqhaaWcbaGaemiuaafabaGaeiikaGIaemiBaWMaeiykaKcaaaaa@3282@(**u**_*s*_), s = 1,..., S} and the corresponding prediction variance {[σP(l)
 MathType@MTEF@5@5@+=feaafiart1ev1aaatCvAUfKttLearuWrP9MDH5MBPbIqV92AaeXatLxBI9gBaebbnrfifHhDYfgasaacH8akY=wiFfYdH8Gipec8Eeeu0xXdbba9frFj0=OqFfea0dXdd9vqai=hGuQ8kuc9pgc9s8qqaq=dirpe0xb9q8qiLsFr0=vr0=vr0dc8meaabaqaciaacaGaaeqabaqabeGadaaakeaaiiGacqWFdpWCdaqhaaWcbaGaemiuaafabaGaeiikaGIaemiBaWMaeiykaKcaaaaa@32DF@(**u**_*s*_)]^2^, s = 1,..., S} were compared to the underlying risk map {r(**u**_s_), s = 1,..., S}. As in a previous study on the performances of alternative smoothing techniques [15], various criteria were computed and averaged over all realizations to attenuate the impact of statistical fluctuations.

#### Bias and accuracy of prediction

The first two criteria are the mean error (ME) and mean absolute error (MAE) of prediction computed as:

ME(l)=1W∑s=1Sωs[rP(l)(us)−r(us)]withW=∑s=1Sωs     (Equation 31)
 MathType@MTEF@5@5@+=feaafiart1ev1aaatCvAUfKttLearuWrP9MDH5MBPbIqV92AaeXatLxBI9gBaebbnrfifHhDYfgasaacH8akY=wiFfYdH8Gipec8Eeeu0xXdbba9frFj0=OqFfea0dXdd9vqai=hGuQ8kuc9pgc9s8qqaq=dirpe0xb9q8qiLsFr0=vr0=vr0dc8meaabaqaciaacaGaaeqabaqabeGadaaakeaafaqabeqadaaabaGaemyta0Kaemyrau0aaWbaaSqabeaacqGGOaakcqWGSbaBcqGGPaqkaaGccqGH9aqpdaWcaaqaaiabigdaXaqaaiabdEfaxbaadaaeWbqaaGGaciab=L8a3naaBaaaleaacqWGZbWCaeqaaaqaaiabdohaZjabg2da9iabigdaXaqaaiabdofatbqdcqGHris5aOWaamWaaeaacqWGYbGCdaqhaaWcbaGaemiuaafabaGaeiikaGIaemiBaWMaeiykaKcaaOGaeiikaGccbeGae4xDau3aaSbaaSqaaiabdohaZbqabaGccqGGPaqkcqGHsislcqWGYbGCcqGGOaakcqGF1bqDdaWgaaWcbaGaem4CamhabeaakiabcMcaPaGaay5waiaaw2faaaqaaiabbEha3jabbMgaPjabbsha0jabbIgaObqaaiabbEfaxjabg2da9maaqahabaGae8xYdC3aaSbaaSqaaiabdohaZbqabaaabaGaem4CamNaeyypa0JaeGymaedabaGaem4uamfaniabggHiLdaaaOGaaCzcaiaaxMaadaqadaqaaiabbweafjabbghaXjabbwha1jabbggaHjabbsha0jabbMgaPjabb+gaVjabb6gaUjabbccaGiabiodaZiabigdaXaGaayjkaiaawMcaaaaa@75BF@

MAE(l)=1W∑s=1Sωs|rP(l)(us)−r(us)|withW=∑s=1Sωs     (Equation 32)
 MathType@MTEF@5@5@+=feaafiart1ev1aaatCvAUfKttLearuWrP9MDH5MBPbIqV92AaeXatLxBI9gBaebbnrfifHhDYfgasaacH8akY=wiFfYdH8Gipec8Eeeu0xXdbba9frFj0=OqFfea0dXdd9vqai=hGuQ8kuc9pgc9s8qqaq=dirpe0xb9q8qiLsFr0=vr0=vr0dc8meaabaqaciaacaGaaeqabaqabeGadaaakeaafaqabeqadaaabaGaemyta0KaemyqaeKaemyrau0aaWbaaSqabeaacqGGOaakcqWGSbaBcqGGPaqkaaGccqGH9aqpdaWcaaqaaiabigdaXaqaaiabdEfaxbaadaaeWbqaaGGaciab=L8a3naaBaaaleaacqWGZbWCaeqaaaqaaiabdohaZjabg2da9iabigdaXaqaaiabdofatbqdcqGHris5aOWaaqWaaeaacqWGYbGCdaqhaaWcbaGaemiuaafabaGaeiikaGIaemiBaWMaeiykaKcaaOGaeiikaGccbeGae4xDau3aaSbaaSqaaiabdohaZbqabaGccqGGPaqkcqGHsislcqWGYbGCcqGGOaakcqGF1bqDdaWgaaWcbaGaem4CamhabeaakiabcMcaPaGaay5bSlaawIa7aaqaaiabbEha3jabbMgaPjabbsha0jabbIgaObqaaiabbEfaxjabg2da9maaqahabaGae8xYdC3aaSbaaSqaaiabdohaZbqabaaabaGaem4CamNaeyypa0JaeGymaedabaGaem4uamfaniabggHiLdaaaOGaaCzcaiaaxMaadaqadaqaaiabbweafjabbghaXjabbwha1jabbggaHjabbsha0jabbMgaPjabb+gaVjabb6gaUjabbccaGiabiodaZiabikdaYaGaayjkaiaawMcaaaaa@77FC@

The prediction error at each grid node ***u***_s _is weighted according to the population size at that location, *ω*_s _= n(***u***_s_), in order to penalize more the errors that affect a larger population. Table 2 (top rows) indicates that, on average over 100 realizations, all prediction methods are relatively unbiased. However, in particular for cervix, the smallest bias is the most frequently (i.e. 51% of realizations) observed for ATP kriging estimates. Methods differ much more in terms of the mean absolute error of prediction; see Table 2 (bottom rows). On average, ATP kriging reduces the absolute error of simple prediction methods by 20% for cervix and 5–10% for lung. The benefit of Poisson kriging over kriging of raw or empirical Bayesian smoothed rates is almost systematic since it leads to smaller MAE values 99% of the time for cervix and 89% for lung cancer. Although the deconvoluted semivariogram models depart somewhat from the true point-support models (recall Figure 12, left column), the use of the true model *γ*_R_(**h**) (black curve in Figure 12, left column) in ATP kriging does not cause a substantial decline in prediction errors; compare the last two rows of Table 2 for each performance criterion.

**Table 2:** Performance comparison of alternative kriging estimators: mean errors and mean absolute errors of prediction.

Estimators	Lung cancer		Cervix cancer	
MEAN ERROR	Average	% best result	Average	% best result

Point kriging of raw rates	-0.013	41	0.060	11
Point kriging of global EBS	**0.008**	11	0.040	15
Point kriging of local EBS	0.042	8	0.044	23
ATP Poisson kriging	-0.036	40	**-0.001**	51
ATP Poisson kriging (true *γ*_R_(**h**))	-0.032		-0.001	

MEAN ABSOLUTE ERROR				

Point kriging of raw rates	2.647	8	0.406	0
Point kriging of global EBS	2.694	3	0.406	1
Point kriging of local EBS	2.776	0	0.418	0
ATP Poisson kriging	**2.471**	89	**0.317**	99
ATP Poisson kriging (true *γ*_R_(**h**))	2.452		0.313	

Results obtained on average over 100 realizations generated for Regions 1 and 2. Poisson kriging was conducted with the semivariogram model derived through deconvolution or inferred directly from the simulated grid risk values (true point-support model *γ*_R_(**h**)). Bold numbers refer to best performances outside the ideal case where the true semivariogram of risk is known. The second column gives the percentage of realizations where the particular method (except ATP kriging with true *γ*_R_(**h**)) yields the smallest prediction error.

#### Variance of the prediction errors and smoothing effect

In addition to a risk estimate, all interpolation methods provide a measure of the uncertainty attached to this estimate in the form of the kriging variance (Equations 12 and 23). For the point kriging approaches, the variance [σP(l)
 MathType@MTEF@5@5@+=feaafiart1ev1aaatCvAUfKttLearuWrP9MDH5MBPbIqV92AaeXatLxBI9gBaebbnrfifHhDYfgasaacH8akY=wiFfYdH8Gipec8Eeeu0xXdbba9frFj0=OqFfea0dXdd9vqai=hGuQ8kuc9pgc9s8qqaq=dirpe0xb9q8qiLsFr0=vr0=vr0dc8meaabaqaciaacaGaaeqabaqabeGadaaakeaaiiGacqWFdpWCdaqhaaWcbaGaemiuaafabaGaeiikaGIaemiBaWMaeiykaKcaaaaa@32DF@(**u**_*s*_)]^2 ^depends on the proximity of county centroids (i.e. center of mass of population within the county) to the grid node ***u***_s_, as well as the form of the semivariogram model (i.e. nugget effect, range of autocorrelation). The ATP kriging variance directly accounts for the population size at the county and grid levels. For each interpolation method, the kriging variance was averaged over all *S *grid nodes, resulting in the following statistic:

VPE(l)=1S∑s=1S[σP(l)(us)]2     (Equation 33)
 MathType@MTEF@5@5@+=feaafiart1ev1aaatCvAUfKttLearuWrP9MDH5MBPbIqV92AaeXatLxBI9gBaebbnrfifHhDYfgasaacH8akY=wiFfYdH8Gipec8Eeeu0xXdbba9frFj0=OqFfea0dXdd9vqai=hGuQ8kuc9pgc9s8qqaq=dirpe0xb9q8qiLsFr0=vr0=vr0dc8meaabaqaciaacaGaaeqabaqabeGadaaakeaacqqGwbGvcqqGqbaucqqGfbqrdaahaaWcbeqaaiabcIcaOiabdYgaSjabcMcaPaaakiabg2da9maalaaabaGaeGymaedabaGaem4uamfaamaaqahabaWaamWaaeaaiiGacqWFdpWCdaqhaaWcbaGaemiuaafabaGaeiikaGIaemiBaWMaeiykaKcaaOGaeiikaGccbeGae4xDau3aaSbaaSqaaiabdohaZbqabaGccqGGPaqkaiaawUfacaGLDbaaaSqaaiabdohaZjabg2da9iabigdaXaqaaiabdofatbqdcqGHris5aOWaaWbaaSqabeaacqaIYaGmaaGccaWLjaGaaCzcamaabmaabaGaeeyrauKaeeyCaeNaeeyDauNaeeyyaeMaeeiDaqNaeeyAaKMaee4Ba8MaeeOBa4MaeeiiaaIaeG4mamJaeG4mamdacaGLOaGaayzkaaaaaa@5BDD@

For both cancers, the smallest kriging variance is obtained for point kriging of smoothed (EBS) rates (see Table 3), which is expected because of the lower sill of the corresponding semivariogram models; recall examples of Figure 12 (right column). Conversely, the noise caused by the small number problem leads to higher sills for the semivariograms of raw rates, hence larger kriging variances, in particular for cervix cancer.

**Table 3:** Performance comparison of alternative kriging estimators: kriging variance and smoothing effect.

Estimators	Lung cancer		Cervix cancer	
	Kriging variance	Dispersion variance	Kriging variance	Dispersion variance

Point kriging of raw rates	9.691	5.536	2.724	1.726
Point kriging of global EBS	3.423	2.934	0.314	0.281
Point kriging of local EBS	3.307	2.902	0.237	0.448
ATP Poisson kriging	9.378	8.401	0.645	0.926
ATP Poisson kriging (true *γ*_R_(**h**))	9.470	8.654	0.849	1.089

Results obtained on average over 100 realizations generated for Regions 1 and 2. The dispersion variance refers to the variance of risk estimates. Poisson kriging was conducted with the semivariogram model derived through deconvolution or inferred directly from the simulated grid risk values (true point-support model *γ*_R_(**h**)).

The semivariogram model not only influences the magnitude of the kriging variance at each grid node ***u***_s_, but it also controls the smoothness of the maps of risk estimates. The variance of the risk estimates was computed for each realization and the average over 100 realizations is listed in Table 3. The variance of the reference risk values, averaged over the five scenarios for the risk map, is 18.137 and 1.828 for lung and cervix cancers, respectively. In agreement with the results displayed for one realization in Figures 13 and 14, point kriging of empirical Bayes estimates generates the largest smoothing effect: the variance represents 15–25% of the reference risk variance. The smoothing effect of ATP kriging is much smaller and similar for both cancers: the ratio of variances is 47–50%. Results for point kriging of raw rates are strongly affected by the reliability of the original data: the smoothing is much smaller when interpolating the unreliable cervix mortality rates (ratio = 94%) than for lung cancer (ratio = 31%).

#### Quality of the uncertainty model

According to the results of Table 3, point kriging of EBS rates yields the most accurate prediction since the corresponding kriging variance is the smallest; a conclusion that conflicts with results of Table 2. The ability of the prediction variance to capture the actual magnitude of the prediction error was quantified using the following mean square standardized residual (MSSR):

MSSR(l)=1S∑s=1S[rP(l)(us)−r(us)σP(l)(us)]2     (Equation 34)
 MathType@MTEF@5@5@+=feaafiart1ev1aaatCvAUfKttLearuWrP9MDH5MBPbIqV92AaeXatLxBI9gBaebbnrfifHhDYfgasaacH8akY=wiFfYdH8Gipec8Eeeu0xXdbba9frFj0=OqFfea0dXdd9vqai=hGuQ8kuc9pgc9s8qqaq=dirpe0xb9q8qiLsFr0=vr0=vr0dc8meaabaqaciaacaGaaeqabaqabeGadaaakeaacqWGnbqtcqWGtbWucqWGtbWucqWGsbGudaahaaWcbeqaaiabcIcaOiabdYgaSjabcMcaPaaakiabg2da9maalaaabaGaeGymaedabaGaem4uamfaamaaqahabaWaamWaaeaadaWcaaqaaiabdkhaYnaaDaaaleaacqWGqbauaeaacqGGOaakcqWGSbaBcqGGPaqkaaGccqGGOaakieqacqWF1bqDdaWgaaWcbaGaem4CamhabeaakiabcMcaPiabgkHiTiabdkhaYjabcIcaOiab=vha1naaBaaaleaacqWGZbWCaeqaaOGaeiykaKcabaacciGae43Wdm3aa0baaSqaaiabdcfaqbqaaiabcIcaOiabdYgaSjabcMcaPaaakiabcIcaOiab=vha1naaBaaaleaacqWGZbWCaeqaaOGaeiykaKcaaaGaay5waiaaw2faaaWcbaGaem4CamNaeyypa0JaeGymaedabaGaem4uamfaniabggHiLdGcdaahaaWcbeqaaiabikdaYaaakiaaxMaacaWLjaWaaeWaaeaacqqGfbqrcqqGXbqCcqqG1bqDcqqGHbqycqqG0baDcqqGPbqAcqqGVbWBcqqGUbGBcqqGGaaicqaIZaWmcqaI0aanaiaawIcacaGLPaaaaaa@6EF8@

If the actual estimation error is equal, on average, to the error predicted by the model, the MSSR statistic should be about one [[31], p. 91]. The MSSR statistic was averaged across the *L *realizations according to the following formula:

MSSR=1L∑l=1L[δlMSSR(l)+(1−δl)MSSR(l)]     (Equation 35)
 MathType@MTEF@5@5@+=feaafiart1ev1aaatCvAUfKttLearuWrP9MDH5MBPbIqV92AaeXatLxBI9gBaebbnrfifHhDYfgasaacH8akY=wiFfYdH8Gipec8Eeeu0xXdbba9frFj0=OqFfea0dXdd9vqai=hGuQ8kuc9pgc9s8qqaq=dirpe0xb9q8qiLsFr0=vr0=vr0dc8meaabaqaciaacaGaaeqabaqabeGadaaakeaacqWGnbqtcqWGtbWucqWGtbWucqWGsbGucqGH9aqpdaWcaaqaaiabigdaXaqaaiabdYeambaadaaeWbqaamaadmaabaacciGae8hTdq2aaSbaaSqaaiabdYgaSbqabaGccqWGnbqtcqWGtbWucqWGtbWucqWGsbGudaahaaWcbeqaaiabcIcaOiabdYgaSjabcMcaPaaakiabgUcaRmaalaaabaGaeiikaGIaeGymaeJaeyOeI0Iae8hTdq2aaSbaaSqaaiabdYgaSbqabaGccqGGPaqkaeaacqWGnbqtcqWGtbWucqWGtbWucqWGsbGudaahaaWcbeqaaiabcIcaOiabdYgaSjabcMcaPaaaaaaakiaawUfacaGLDbaaaSqaaiabdYgaSjabg2da9iabigdaXaqaaiabdYeambqdcqGHris5aOGaaCzcaiaaxMaadaqadaqaaiabbweafjabbghaXjabbwha1jabbggaHjabbsha0jabbMgaPjabb+gaVjabb6gaUjabbccaGiabiodaZiabiwda1aGaayjkaiaawMcaaaaa@6866@

with *δ*_l _= 1 if MSSR^(l)^>1, and zero otherwise. This averaging allows one to penalize equally the over- and under-estimation of the prediction errors by the kriging variance. Table 4 (top rows) indicates that the EBS kriging variance fails to inform on the actual magnitude of the prediction errors. Because *δ*_l _= 1 for all realizations, the MSSR statistic means that, on average over all realizations, the actual squared prediction error is 4 to 7 times larger than what is predicted using the kriging variance. This over-optimistic assessment of the performance of EBS kriging results from the smoothing of rates in the empirical Bayes approach. For lung cancer, point kriging of raw rates yields the best results most of the times (i.e. 79% of realizations). However, this result simply indicates that one correctly predicts that observed rates fare badly in estimating the underlying risk (recall Table 2). This approach does not perform as well for least frequent cancers, such as cervix cancer, where the lack of reliability of rates is not captured by the analysis. Because semivariogram estimation is very sensitive to extreme unreliable rates, the sill of the semivariogram model for raw rates greatly varies among the 100 realizations, leading to a wide range of MSSR values: 0.22–13. This lack of robustness prevents the identification of over or under-estimation of the prediction errors in actual applications. Poisson kriging is much more robust with respect to the type of cancer and leads to similar average MSSR for lung and cervix. The range of MSSR for cervix is also much narrower (0.75–4.36), which indicates a better quantification of the magnitude of prediction errors by the kriging variance. The fact that the best results overall are obtained by far for ATP kriging with the true semivariogram model (e.g., the range of cervix MSSR is 0.75–1.57) suggests that future improvements of the method should focus on the estimation of the point-support semivariogram model.

**Table 4:** Performance comparison of alternative kriging estimators: mean square standardized residual and goodness of uncertainty models.

Estimators	Lung cancer		Cervix cancer	
MSSR	Average	% best result	Average	% best result

Point kriging of raw rates	**1.707**	79	2.176	41
Point kriging of global EBS	5.941	0	4.566	0
Point kriging of local EBS	6.310	0	6.599	0
ATP Poisson kriging	1.827	21	**1.776**	59
ATP Poisson kriging (true *γ*_R_(**h**))	1.081		1.086	

GOODNESS STATISTIC				

Point kriging of raw rates	**0.918**	78	0.868	27
Point kriging of global EBS	0.602	0	0.731	3
Point kriging of local EBS	0.580	0	0.616	0
ATP Poisson kriging	0.862	22	**0.916**	70
ATP Poisson kriging (true *γ*_R_(**h**))	0.981		0.959	

Results obtained on average over 100 realizations generated for Regions 1 and 2. Poisson kriging was conducted with the semivariogram model derived through deconvolution or inferred directly from the simulated grid risk values (true point-support model *γ*_R_(**h**)). Bold numbers refer to best performances (i.e. criteria closer to 1) outside the ideal case where the true semivariogram of risk is known. The second column gives the percentage of realizations where the particular method (except ATP kriging with true *γ*_R_(**h**)) yields the best results.

Another way to use the prediction variance is to build at each grid node ***u***_s _the conditional cumulative distribution function (ccdf) of the unknown risk value. Under the assumption of normality of the prediction errors, the ccdf is fully characterized by its mean and variance which are the risk estimate, rP(l)
 MathType@MTEF@5@5@+=feaafiart1ev1aaatCvAUfKttLearuWrP9MDH5MBPbIqV92AaeXatLxBI9gBaebbnrfifHhDYfgasaacH8akY=wiFfYdH8Gipec8Eeeu0xXdbba9frFj0=OqFfea0dXdd9vqai=hGuQ8kuc9pgc9s8qqaq=dirpe0xb9q8qiLsFr0=vr0=vr0dc8meaabaqaciaacaGaaeqabaqabeGadaaakeaacqWGYbGCdaqhaaWcbaGaemiuaafabaGaeiikaGIaemiBaWMaeiykaKcaaaaa@3282@(**u**_*s*_), and the prediction variance, [σP(l)
 MathType@MTEF@5@5@+=feaafiart1ev1aaatCvAUfKttLearuWrP9MDH5MBPbIqV92AaeXatLxBI9gBaebbnrfifHhDYfgasaacH8akY=wiFfYdH8Gipec8Eeeu0xXdbba9frFj0=OqFfea0dXdd9vqai=hGuQ8kuc9pgc9s8qqaq=dirpe0xb9q8qiLsFr0=vr0=vr0dc8meaabaqaciaacaGaaeqabaqabeGadaaakeaaiiGacqWFdpWCdaqhaaWcbaGaemiuaafabaGaeiikaGIaemiBaWMaeiykaKcaaaaa@32DF@(**u**_*s*_)]^2^. The probability that the risk variable does not exceed any specific threshold *r *at ***u***_s _is then:

FR(l)(us;r|(Info))=Prob{R(us)≤r|(Info)}=G[(r−rP(l)(us))/σP(l)(us)]     (Equation 36)
 MathType@MTEF@5@5@+=feaafiart1ev1aaatCvAUfKttLearuWrP9MDH5MBPbIqV92AaeXatLxBI9gBaebbnrfifHhDYfgasaacH8akY=wiFfYdH8Gipec8Eeeu0xXdbba9frFj0=OqFfea0dXdd9vqai=hGuQ8kuc9pgc9s8qqaq=dirpe0xb9q8qiLsFr0=vr0=vr0dc8meaabaqaciaacaGaaeqabaqabeGadaaakeaafaqaceGabaaabaGaemOray0aa0baaSqaaiabdkfasbqaaiabcIcaOiabdYgaSjabcMcaPaaakiabcIcaOGqabiab=vha1naaBaaaleaacqWGZbWCaeqaaOGaei4oaSJaemOCaiNaeiiFaWNaeiikaGIaeeysaKKaeeOBa4MaeeOzayMaee4Ba8MaeiykaKIaeiykaKIaeyypa0JaeeiuaaLaeeOCaiNaee4Ba8MaeeOyaiMaei4EaSNaemOuaiLaeiikaGIae8xDau3aaSbaaSqaaiabdohaZbqabaGccqGGPaqkcqGHKjYOcqWGYbGCcqGG8baFcqGGOaakcqqGjbqscqqGUbGBcqqGMbGzcqqGVbWBcqGGPaqkcqGG9bqFaeaacqGH9aqpcqWGhbWrdaWadaqaamaabmaabaGaemOCaiNaeyOeI0IaemOCai3aa0baaSqaaiabdcfaqbqaaiabcIcaOiabdYgaSjabcMcaPaaakiabcIcaOiab=vha1naaBaaaleaacqWGZbWCaeqaaOGaeiykaKcacaGLOaGaayzkaaGaei4la8ccciGae43Wdm3aa0baaSqaaiabdcfaqbqaaiabcIcaOiabdYgaSjabcMcaPaaakiabcIcaOiab=vha1naaBaaaleaacqWGZbWCaeqaaOGaeiykaKcacaGLBbGaayzxaaaaaiaaxMaacaWLjaWaaeWaaeaacqqGfbqrcqqGXbqCcqqG1bqDcqqGHbqycqqG0baDcqqGPbqAcqqGVbWBcqqGUbGBcqqGGaaicqaIZaWmcqaI2aGnaiaawIcacaGLPaaaaaa@8B70@

where *G*(·) is the cumulative distribution function of the standard normal distribution. The ccdf allows the computation of symmetric *p*-probability intervals (PI) centred on the median; for example, the 0.5-PI is bounded by the lower and upper quartiles [FR−1
 MathType@MTEF@5@5@+=feaafiart1ev1aaatCvAUfKttLearuWrP9MDH5MBPbIqV92AaeXatLxBI9gBaebbnrfifHhDYfgasaacH8akY=wiFfYdH8Gipec8Eeeu0xXdbba9frFj0=OqFfea0dXdd9vqai=hGuQ8kuc9pgc9s8qqaq=dirpe0xb9q8qiLsFr0=vr0=vr0dc8meaabaqaciaacaGaaeqabaqabeGadaaakeaacqWGgbGrdaqhaaWcbaGaemOuaifabaGaeyOeI0IaeGymaedaaaaa@30F8@(**u**_s_;0.25|(Info)), FR−1
 MathType@MTEF@5@5@+=feaafiart1ev1aaatCvAUfKttLearuWrP9MDH5MBPbIqV92AaeXatLxBI9gBaebbnrfifHhDYfgasaacH8akY=wiFfYdH8Gipec8Eeeu0xXdbba9frFj0=OqFfea0dXdd9vqai=hGuQ8kuc9pgc9s8qqaq=dirpe0xb9q8qiLsFr0=vr0=vr0dc8meaabaqaciaacaGaaeqabaqabeGadaaakeaacqWGgbGrdaqhaaWcbaGaemOuaifabaGaeyOeI0IaeGymaedaaaaa@30F8@(**u**_s_;0.75|(Info))]. A correct modeling of local uncertainty would entail that there is a 0.5 probability that the actual risk value at **u**_s _falls into that interval or, equivalently, that over the study area 50% of the 0.5-PI include the true value. Since the true risk maps are known for our simulation studies, the fraction of true values falling into any given *p*-PI, denoted ζ¯
 MathType@MTEF@5@5@+=feaafiart1ev1aaatCvAUfKttLearuWrP9MDH5MBPbIqV92AaeXatLxBI9gBaebbnrfifHhDYfgasaacH8akY=wiFfYdH8Gipec8Eeeu0xXdbba9frFj0=OqFfea0dXdd9vqai=hGuQ8kuc9pgc9s8qqaq=dirpe0xb9q8qiLsFr0=vr0=vr0dc8meaabaqaciaacaGaaeqabaqabeGadaaakeaaiiGacuWF2oGEgaqeaaaa@2E88@^(*l*)^(*p*), can be computed easily and compared to the expected fraction *p*. Following Deutsch [32], the agreement between estimated and theoretical fractions is quantified using the following "goodness" statistic:

G(l)=1−1K′∑k=1K′w(pk)|ζ¯(l)(pk)−pk|with0≤G(l)≤1     (Equation 37)
 MathType@MTEF@5@5@+=feaafiart1ev1aaatCvAUfKttLearuWrP9MDH5MBPbIqV92AaeXatLxBI9gBaebbnrfifHhDYfgasaacH8akY=wiFfYdH8Gipec8Eeeu0xXdbba9frFj0=OqFfea0dXdd9vqai=hGuQ8kuc9pgc9s8qqaq=dirpe0xb9q8qiLsFr0=vr0=vr0dc8meaabaqaciaacaGaaeqabaqabeGadaaakeaafaqabeqadaaabaGaem4raC0aaWbaaSqabeaacqGGOaakcqWGSbaBcqGGPaqkaaGccqGH9aqpcqaIXaqmcqGHsisldaWcaaqaaiabigdaXaqaaiqbdUealzaafaaaamaaqahabaGaem4DaCNaeiikaGIaemiCaa3aaSbaaSqaaiabdUgaRbqabaGccqGGPaqkcqGG8baFiiGacuWF2oGEgaqeamaaCaaaleqabaGaeiikaGIaemiBaWMaeiykaKcaaOGaeiikaGIaemiCaa3aaSbaaSqaaiabdUgaRbqabaGccqGGPaqkcqGHsislcqWGWbaCdaWgaaWcbaGaem4AaSgabeaakiabcYha8bWcbaGaem4AaSMaeyypa0JaeGymaedabaGafm4saSKbauaaa0GaeyyeIuoaaOqaaiabbEha3jabbMgaPjabbsha0jabbIgaObqaaiabicdaWiabgsMiJkabdEeahnaaCaaaleqabaGaeiikaGIaemiBaWMaeiykaKcaaOGaeyizImQaeGymaedaaiaaxMaacaWLjaWaaeWaaeaacqqGfbqrcqqGXbqCcqqG1bqDcqqGHbqycqqG0baDcqqGPbqAcqqGVbWBcqqGUbGBcqqGGaaicqaIZaWmcqaI3aWnaiaawIcacaGLPaaaaaa@7363@

where *w(p_*k*_) *= 1 if ζ¯
 MathType@MTEF@5@5@+=feaafiart1ev1aaatCvAUfKttLearuWrP9MDH5MBPbIqV92AaeXatLxBI9gBaebbnrfifHhDYfgasaacH8akY=wiFfYdH8Gipec8Eeeu0xXdbba9frFj0=OqFfea0dXdd9vqai=hGuQ8kuc9pgc9s8qqaq=dirpe0xb9q8qiLsFr0=vr0=vr0dc8meaabaqaciaacaGaaeqabaqabeGadaaakeaaiiGacuWF2oGEgaqeaaaa@2E88@^(*l*)^(*p*_*k*_) >*p*_*k*_, and 2 otherwise. The weights *w *penalize the situation where the fraction of true values falling into the *p*-PI is smaller than expected. K' represents the discretization level of the computation; for example, the ccdf percentiles are used as PI boundaries when K' = 50. The goodness statistic confirms the ranking of methods obtained for MSSR; see Table 4 (bottom rows). Point kriging of EBS estimates yields the worst results, while Poisson kriging with the true semivariogram model outperforms other methods. Poisson kriging with the deconvoluted semivariogram model is the 2^nd ^best for the less frequent cervix cancer. Point kriging of raw rates performs better for cancer with high incidence since the corresponding mortality rates are less unstable.

## Conclusion

Capitalizing on recent work in the area of change of support and semivariogram deconvolution, this paper generalized the Poisson kriging approach in order to incorporate not only the shape and size of geographical units, but also the spatial repartition of population within those units in the filtering of cancer mortality maps. The new formulation can accommodate any measurement or prediction support, enabling the estimation of mortality risk both at the level of administrative units (area-to-area kriging) or at the nodes of a fine grid discretizing the area of interest (area-to-point kriging). Unlike the empirical geostatistical methods recently proposed for the creation of isopleth maps from areal cancer rates, the present methodology: 1) conducts the filtering and mapping in a single step, 2) ensures the coherence of the prediction, that is the population-weighted average of kriged risks within each geographical unit equals the risk for this unit, and 3) provides a measure of uncertainty (i.e. kriging variance) that accounts for the spatial pattern of mortality risk, the geometry of administrative units and the spatial repartition of the population at risk.

The analysis of age-adjusted lung and cervix cancer mortality rates illustrated some key features of area-to-point (ATP) Poisson kriging relatively to point kriging of raw rates or empirical Bayesian smoothed rates. For both cancers, the coherence constraint implicit to ATP kriging attenuates the smoothness of the kriged maps while the incorporation of the high-resolution population map enhances the impact of low or high rates recorded in the vicinity of urban areas. Because point kriging of areal data arbitrarily assigns the entire county population to the location of its centroid, information about the size and shape of the geographical unit is lost. Point kriging risk maps can either display unrealistically large variability if based on unreliable raw mortality rates or can be over-smoothed if rates are first filtered using an empirical Bayes approach. Differences between prediction methods are even more pronounced with respect to the prediction variance. The point kriging variance is mainly influenced by the location of county centroids: low values simply indicate the presence of clusters of small counties, while large values are observed close to the edges of the study area. A unique feature of the ATP kriging variance is its incorporation of population sizes, leading to lower prediction variance around urban centres. Although the use of population-weighted versus geographic centroids improved the point kriging predictions (results not shown), ATP kriging yields the most accurate predictions for both cancers. Simulation studies also showed that the ATP kriging variance provides a better quantification of the magnitude of prediction errors. The benefit of the proposed approach is the largest for cervix cancer measured in Region 2 because of the combined effect of the low reliability of mortality rates and the wide range of county shapes and sizes.

In earlier papers on the use of Poisson kriging for filtering noise in cancer mortality maps [15], a point kriging approach was implemented whereby only county geographic centroids were considered for semivariogram estimation and kriging. Besides prohibiting the disaggregation of areal data, point Poisson kriging overestimates the prediction variance associated with the areal estimates. The trade-off costs for the implementation of the new form of Poisson kriging are: 1) the inference of the point-support semivariogram from the semivariogram of areal data, 2) the CPU time associated with the computation of area-to-area or area-to-point covariance terms through the fine discretization of geographical units. This paper introduced an innovative deconvolution procedure that allows such an inference in presence of irregular area and heterogeneous population density. Simulation studies demonstrated the convergence of the iterative approach that yields theoretically regularized semivariogram models that are very close to the model inferred from areal data. Although the deconvoluted semivariogram models depart somewhat from the true point-support models, the estimation of risk values appeared to be fairly robust with respect of misspecifications of the semivariogram model. Better prediction of the semivariogram sill would however benefit the quality of the models of uncertainty provided by Poisson kriging. Computational time can be substantially reduced by the use of coarser discretizing grids (e.g. 10 km spacing instead of 5 km). Sensitivity analysis [10] indicates that the minor changes in the deconvoluted model caused by the use of those coarser grids bear little consequences on the kriging predictions.

In their common implementation, full Bayes models fail to account for the size and shape of administrative units, as well as the distribution of the population at risk within those units [17,18]. Simplistic spatial models, such as the popular conditional auto-regressive model, prohibit any change of support and the isopleth mapping of risk values. Therefore, ATP Poisson kriging could only be compared to point kriging of raw rates or empirical Bayesian smoothed rates. Although the latter was introduced as a method for "exploratory" disease mapping [3], it is based on kriging and semivariogram modelling and represents a valid yardstick for the comparison study. When conducting spatial interpolation, one should never forget that every measurement relates to a non-zero finite sample volume. Whereas it is customary in earth science applications to assimilate that measurement support to a point, such a practice becomes a crude simplification for lattice data. Furthermore, treating areal data as point data, then using them to conduct kriging within their own areal support (i.e. disaggregation), is meaningless.

This paper presents a coherent spatial methodology that allows both the filtering of the noise caused by the small number problem and the creation of isopleth maps of mortality risk, thereby avoiding the visual bias associated with the interpretation of choropleth cancer mortality maps. By enabling the estimation of mortality risk over any given support, the approach should facilitate the analysis of relationships between health data and putative covariates (i.e. environmental, socio-economic, behavioral or demographic factors) that are typically measured over different spatial supports. These covariates could also be used directly as secondary information in area-to-point kriging, leading to more detailed risk maps at finer scale [12].

## Competing interests

The author is affiliated with BioMedware a research company that also develops software for the exploratory spatial and temporal analysis of health and environmental data. With funding from the National Cancer Institute, the author developed STIS (Space-Time Intelligence System), which is a commercial product of Terraseer and should include a Poisson kriging function in a future release.
